# Carbonate Reservoir Quality Variations in Basins with a Variable Sediment Influx: A Case Study from the Balkassar Oil Field, Potwar, Pakistan

**DOI:** 10.1021/acsomega.2c06773

**Published:** 2023-01-19

**Authors:** Muhammad Raiees Amjad, Muhsan Ehsan, Muyyassar Hussain, Nadhir Al-Ansari, Abdul Rehman, Zohaib Naseer, Muhammad Nauman Ejaz, Rafik Baouche, Ahmed Elbeltagi

**Affiliations:** † Department of Earth and Environmental Sciences, Bahria School of Engineering and Applied Sciences, Bahria University, Islamabad44000, Pakistan; ‡ Institute of Geophysics and Geomatics, China University of Geosciences, Wuhan430074, P. R. China; § Advance Reservoir Characterization, LMK Resources, Islamabad44000, Pakistan; ∥ Department of Civil, Environmental and Natural Resources Engineering, Lulea University of Technology, Lulea97187, Sweden; ⊥ Department of Geophysics, Laboratory of Resources Minérals at Energétiques, Faculty of Sciences, M’Hamed Bougara University of Boumerdes, Boumerdès35000, Algeria; # Agricultural Engineering Dept., Faculty of Agriculture, Mansoura University, Mansoura35516, Egypt

## Abstract

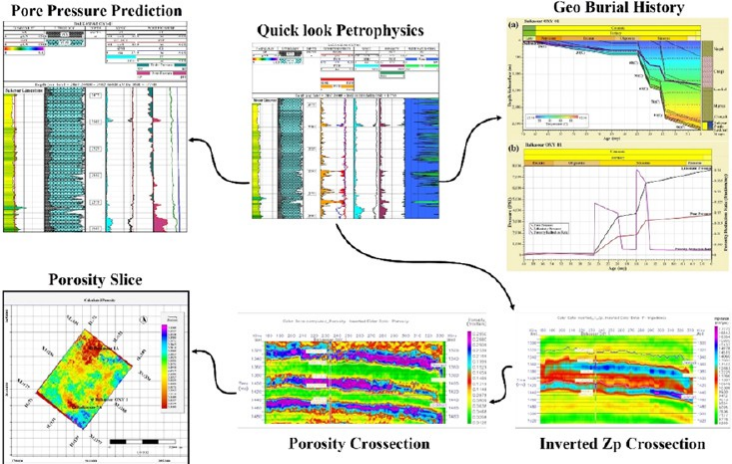

The carbonate reservoir quality is strongly reliant on the compaction process during sediment burial and other processes such as cementation and dissolution. Porosity and pore pressure are the two main factors directly affected by mechanical and chemical compactions. Porosity reduction in these carbonates is critically dependent on the overburden stress and subsidence rate. A variable sediment influx in younger basins may lead to changes in the reservoir quality in response to increasing lithostatic pressure. Deposition of molasse sediments as a result of the Himalayan orogeny caused variations in the sedimentation influx in the Potwar Basin of Pakistan throughout the Neogene times. The basic idea of this study is to analyze the carbonate reservoir quality variations induced by the compaction and variable sediment influx. The Sakesar Limestone of the Eocene age, one of the proven carbonate reservoirs in the Potwar Basin, shows significant changes in the reservoir quality, specifically in terms of porosity and pressure. A 3D seismic cube (10 km^2^) and three wells of the Balkassar field are used for this analysis. To determine the vertical and lateral changes of porosity in the Balkassar area, porosity is computed from both the log and seismic data. The results of both the data sets indicate 2–4% porosities in the Sakesar Limestone. The porosity reduction rate with respect to the lithostatic pressure computed with the help of geohistory analysis represents a sharp decrease in porosity values during the Miocene times. Pore pressure predictions in the Balkassar OXY 01 well indicate underpressure conditions in the Sakesar Limestone. The Eocene limestones deposited before the collision of the Indian plate had enough time for fluid expulsion and show underpressure conditions with high porosities.

## 1 Introduction

The reservoir quality of carbonate rocks is greatly dependent on the compaction process during sediment burial, in addition to other processes such as cementation, dissolution, diagenesis, and depositional microfacies.^1−3^ Porosity reduction with an increase in burial depth of the sedimentary rocks is primarily governed by either mechanical or chemical compaction.^4,5^ As the overburden pressure is increased, the chemical and physical properties of sedimentary rocks begin to change.^6^ Apart from the porosity reduction, the development of pore spaces with time and depth directly impacts subsidence rates. Compaction of the sediments would have a direct effect on many parameters,^7,8^ which includes (1) depositional geometries, (2) fluid flow and pore pressure, (3) thermal conductivity of all lithologies (except salt), (4) thermal maturity and hydrocarbon generation, and (5) burial and tectonic subsidence analysis.

Mechanical compaction in carbonates is affected by the overburden stress, grain size, and clay content. The grain size in carbonates is related to the biological and physical origin of the carbonate.^9^ Due to the increase in friction, adhesion, and bridging with decreasing grain size, compaction of fine sediments is less effective than for coarse grains.^10^ Heterogeneity in grain size distribution enhances mechanical compaction as well.^11^ In carbonate sediments that are mixed with clays, mechanical compaction is more important in layers containing clays.^12^ This can be explained by two mechanisms. On the one hand, clay particles increase the heterogeneity of the grain size distribution. On the other hand, clay trapped along carbonate grain contacts may prevent healing of these contacts and reduce the friction coefficient, allowing grain sliding.^9^

Porosity is one of the petrophysical parameters, which has a profound impact on reservoir characterization and reserves estimation.^13^ Porosity in the rocks in not only dependent on the depth factor but also dependent on the effective stress.^14^ The process of sediment compaction results in a decrease in the porosity and an increase in the bulk density of the rock.^15^ It cannot be always true that porosity might have an indirect relationship with depth.^14^ In general, there would always be a decrease in the porosity of the rocks as the depth increases; however, in some cases, porosity can increase as well, if the effective stress does not follow the normal trend. This might occur under those conditions where the value of effective stress is less compared to the effective stress value in a normal compaction trend along with compaction.^16^

Postdepositional processes such as diagenesis, dolomitization, and dissolution also play their part in defining the reservoir quality of carbonate rocks.^17,18^ Porosity reduction with increased burial depth is dependent on the magnesium concentration of the pore water.^19,20^ If the formation contains fresh water or brackish water, the loss in porosity will be faster compared to that of the formation which contains more magnesium water in it. There is a broad range of porosity/depth values, especially for carbonates. Near the surface, the porosity of limestones is more compared to that of dolomite, but at a greater depth, the porosity of dolomite is more compared to that of limestones. This can be due to the fact dolomite is more resistant to porosity reducing effects of burial.^21,22^ Similarly, when compared to sandstones at the same depth, the porosity of carbonates is significantly lower.^23^ In carbonates, early porosity reduction at shallow depths may help sustain the resulting porosity in deeper burial.^24^

**Figure 1:**
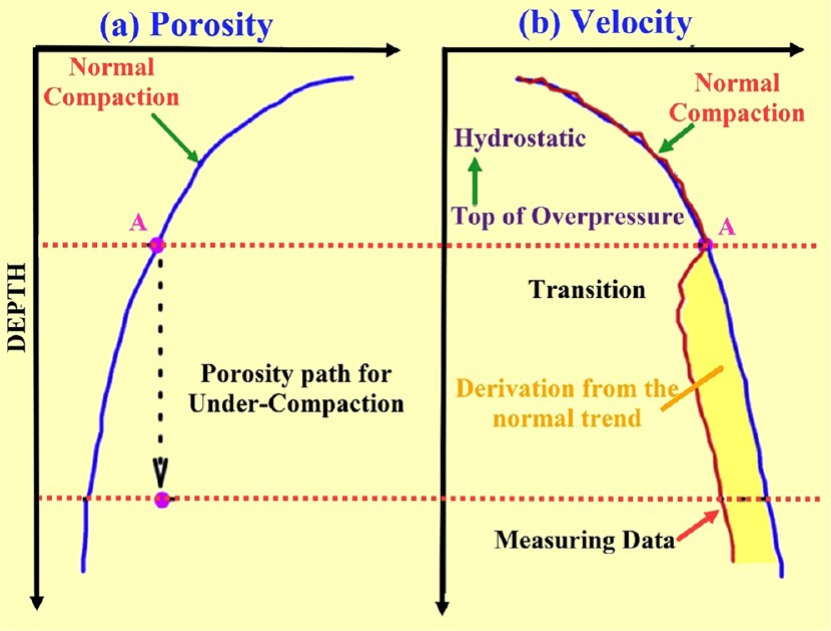
Schematic representation of porosity (a) and velocity (b) with respect to pore pressure variations in overpressured zone. In (a) and (b), the blue curve shows the normal compaction trend with respect to porosity reduction and velocity enhancement, respectively. Point A marks the start of the overpressure zone due to under-compaction of the sediments represented by the black dash line. Velocities in this overpressure interval are lesser than the normal estimated velocities due to high porosities and fluid saturation. This marks the deviation of velocity and porosity curves from the normal compaction trend line (modified from ref (55)).

Compaction is one of the major components, which controls the rock porosity and fluid pressures. It induces progressive reduction in the porosity of rocks in response of the overburden pressure caused by loading of sediments. If the rate of sediment deposition is slow, allowing water in the pore spaces to escape during burial of sediments, the porosity will be low.^25^ When the formation fluid cannot escape through pores at a pace sufficient to maintain equilibrium with the column of formation water, overpressure develops.^26^ Porosity of an overpressure formation will be higher than the estimated porosity under normal compaction, and its respective velocity will be decreased, marking the top of the overpressure zone (Figure 1).

In foreland basins, categorized by molasse depositional sequences, the sediment influx rate plays a critical role in mechanical compaction. According to Law and Shah,^27^ in certain younger Tertiary rocks, overpressure can be caused by the rapid deposition and subsidence of sediments due to vertical loading. Subsidence, compaction, and fluid pressure dynamics can be qualitatively characterized as a competitive process between pressure generation and pressure dissipation.^28^ During the orogenic activity, generation of major thrusts triggers rapid sedimentation in a short span of time. This episodic depositional history of high and low deposition rates significantly affects the mechanical compaction of the underlying carbonate rocks.

The Potwar Basin of Pakistan, an active foreland basin, experienced variable sediment influx during the deposition of Tertiary molasse deposits. In response to this, Eocene carbonate rocks exhibit vertical and spatial variations in terms of their reservoir quality. It is very pertinent to determine the response of porosity and fluid pressures toward variable sediment influx in these active foreland basins. A number of parameters are to be evaluated including facies types, reservoir properties, structural trends, and burial history to determine the compaction trends. In this study, wireline log data of the Balkassar OXY 01 well are used for evaluating the vertical variations of reservoir properties such as porosity, fluid saturation, lithology, and reservoir pressures. This is followed by the determination of the porosity reduction rate and subsidence events in response of the overburden pressure and burial depth. Finally, lateral porosity variations in the Sakesar Limestone are predicted using seismic data with the application of seismic inversion (SI) and artificial neural network (ANN) analysis.

## 2 Geological Settings

**Figure 2:**
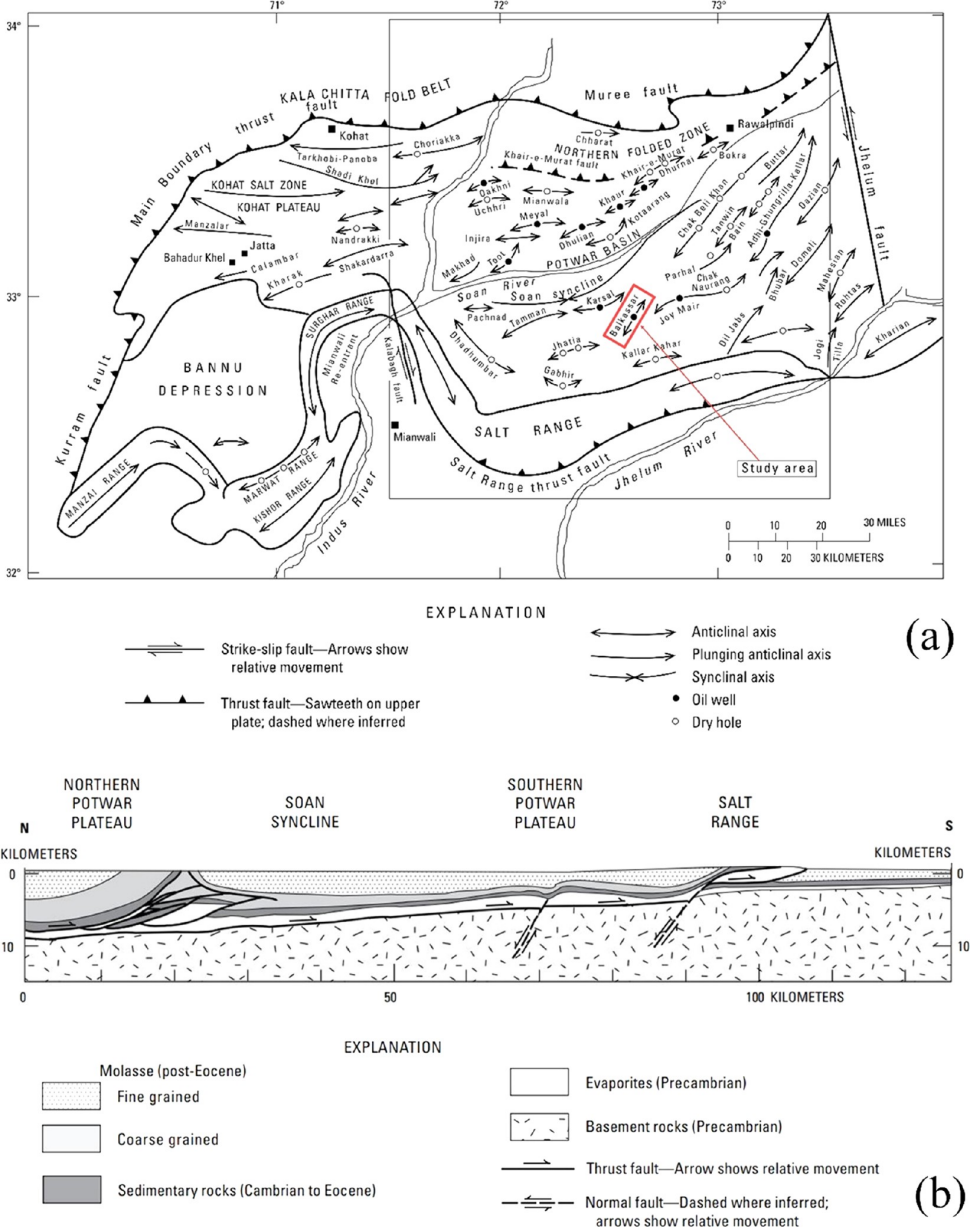
(a) Structural map of the Potwar and Kohat basins, northern Pakistan. The regional trend of the anticlines and synclines in the Potwar Basin follows a northeast–southwest orientation. Balkassar study located in the southern part of the Potwar Basin, highlighted in the red box, also follows the same regional structural trend adopted from refs (29, 32). (b) Generalized cross-section across the western Potwar Basin and the west-central Salt Range. The structural cross-section represents the thickness variation of the pre-Eocene and post-Eocene depositional sequences. The molasse deposits of post-Eocene formations covers the thick top layer of the sedimentary cover, which exerts tremendous overburden pressure on the Eocene carbonate reservoirs of the Potwar Basin. Normal faults are observed in the basement, whereas the thrust faults form a decollement within the evaporites of the Precambrian age representing thin-skinned tectonics in the area.^35,38^

The Balkassar oil field lies in the center of the Potwar Basin, which is one of the major hydrocarbon-producing basins of Pakistan^29^ (Figure 2a). With respect to the sedimentary basins of Pakistan, the Potwar Basin is part of the Upper Indus Basin, and tectonically, its location is marked by the northern and southern margins of sub-Himalayas. The Potwar Basin is bounded in the north by the Main Boundary Thrust (MBT), the Jhelum strike-slip fault in the east, and the Kalabagh strike-slip fault in the west and by the Salt Range Thrust (SRT) in the south.^30,31^ The basin is internally deformed and has a width of 150 km in the north–south direction with an average elevation of 499 m from the sea level.^32^ The Soan syncline, which is a regional synclinal structure present within the basin, divides it into two major parts based on structural deformation styles. Part of the basin toward the north of the Soan syncline is called as the north Potwar deformed zone (NPDZ), whereas the southern part is called as the south Potwar platform zone (SPPZ).^33^

Oil and gas are currently being extracted from the Potwar Basin. Data from different studies^34−37^ of seismic profiles, well logs, Bouguer gravity maps, and surface geology are combined together to constitute the regional structural cross-section, which shows the tectonic features of the area^38^ (Figure 2b). The basin is dominated by several salt-cored anticlines produced by the Himalayan orogeny, which have been targeted for hydrocarbon exploration.^39^ The regional trend of these anticlinal structures is in the NE–SW direction.^40^ The Balkassar oil field is marked by a broad box-shaped anticlinal structure bounded by reverse faults. The oil companies have drilled more wells in the eastern compartment of the Balkassar anticline compared to those in the western compartment.

The Potwar Basin is characterized by complex structural deformations as a result of the tectonic collision between the Indian and Eurasian plates in the early to the middle Eocene age.^41^ SRT, a low-dipping thrust fault, carried the overlying sediments in the southward direction along a Precambrian decollement.^42^ About 150 exploratory wells have been drilled in the area; many were prematurely abandoned because of structural complexity.^40^ Studies conducted elsewhere^43^^44^ suggested that the distributions of porosity and permeability in Eocene carbonates are controlled by depositional, diagenetic, and deformational processes. According to Wrobel-Daveau and Barracloughy,^45^ validation of reservoir properties in the Potwar Basin, particularly for the Eocene Sakesar Limestone, is key to success. Fahad and Khan^46^ mentioned in their study that the reduction in rock volume caused by mechanical compaction potentially deteriorates the carbonate reservoir quality.

The sedimentary succession of the Potwar Basin consists of evaporites, siliciclastic, and carbonate sequences ranging in age from Precambrian to recent (Table 1). The oldest Salt Range formation of the Precambrian age lies directly above the basement rocks.^47^ A regional decollement, SRT, thrusted the overlying stratigraphic sequences including the Jhelum (Cambrian), Nilawahan (Permian), Makarwal (Paleocene), Chharat (Eocene), Rawalpindi (Miocene), and Siwalik (Miocene) groups in the SPPZ.^48^ Shallow marine carbonate deposits of the Eocene age are the last stratigraphic units deposited in the Paleo-Tethys basin before the collision of the Indian Plate.^47^ According to Jadoon and Hinderer,^49^ Eocene carbonates are the major contributor of hydrocarbon production in the Potwar Basin.

**Table 1:** Borehole Stratigraphic Information of the Formations Encountered in Balkassar Wells^a^

age	group	formations	Balkassar OXY 01 (m)	Balkassar 01A (m)	Balkassar 07 (m)
Miocene	Siwaliks	Nagri formation	0	0	0
		Chinji formation	478.8	426.7	455.0
	Rawalpindi	Kamlial formation	1408.1	1426.4	1390.4
		Murree formation	1514.8	1534.7	1497.1
Eocene	Chharat	Chorgali formation	2421.5	2479.5	2398.7
		Sakesar limestone	2467.2	2528.3	2448.1
Paleocene	Makarwal	Patala formation	2602.9	2649.3	2567.3
		Lockhart limestone	2624.2	2677.9	
		Hangu formation	2659.3	2712.7	
Permian	Nilawahan	Sardhai formation	2686.7		
		Warchha sandstone	2796.4		
		Dandot formation	2938.1		
		Tobra formation	2999.1		
Cambrian	Jhelum	Khewra sandstone	3050.9		
Precambrian		Salt Range formation	3129.2		

a The Balkassar OXY 01 well is the deepest drilled well up to the Precambrian Salt Range Formation. Balkassar 01A and Balkassar 07 wells are only drilled up to the Paleocene sequence.

## 3 Materials and Methods

**Figure 3:**
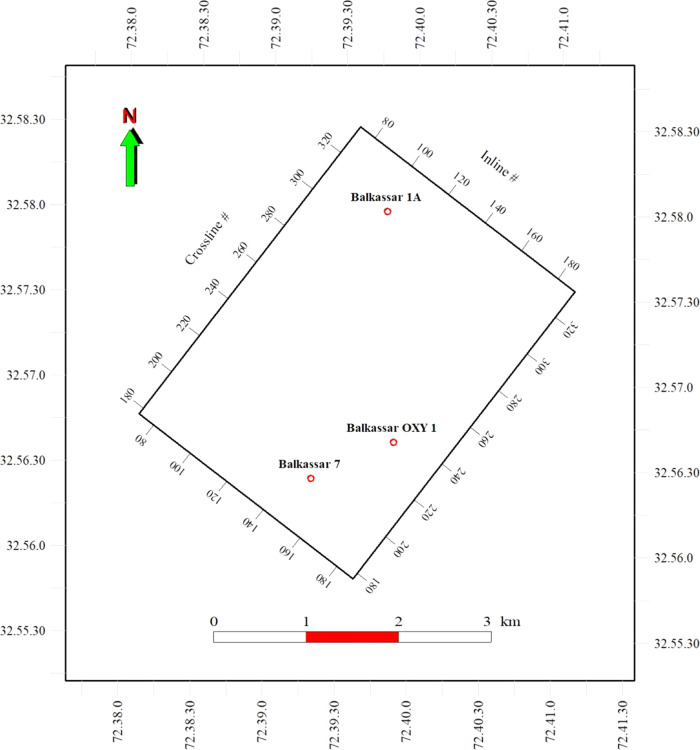
Base map of the Balkassar area showing the boundary of the 3D seismic cube and location of wells. Three wells including Balkassar OXY 01, Balkassar 07, and Balkassar 01 A along with a 10 km^2^ seismic cube covering the central part of the Balkassar anticline are utilized in this study. Crosslines are oriented in the northwest–southeast direction, and inlines are oriented in the northeast–southwest direction.

The current study has been conducted for the pressure and porosity evaluation of the Sakesar Limestone using the 3D seismic cube covering 10 km^2^ of the Balkassar oil field and three wells including Balkassar OXY 01, Balkassar 01A, and Balkassar 07. The base map of the study area showing the orientation of the seismic cube and the location of all three wells is shown in Figure 3. Wireline log data of the wells Balkassar OXY 01, Balkassar 01A, and Balkassar 07 are used for the quick-look petrophysical analysis. The available logs that run in the wells are γ-ray log (GR) spontaneous potential (SP), resistivity (complete suite including LLD, LLS, and MSFL), density (RHOB), sonic (DT), Caliper (CALI), and neutron (PHIN). Subsurface structural interpretation of the Balkassar area is conducted using a 3D seismic cube of the Balkassar block. Check shot data of the Balkassar OXY 01 well are used for the identification of the horizons. Formations encountered in the wells of the Balkassar oil field are shown in Table 1.

### 3.1 Quick-Look Petrophysics

Quick-look petrophysical analysis is performed for the evaluation of basic rock properties including shale volumes, porosities, and fluid saturation of the Sakesar Limestone. The computed porosities are then utilized to predict seismic porosities with the help of ANN. The GR log is used to calculate the reservoir clay volume, as the amount of clay present in a reservoir reduces its permeability. The formula used for calculating the volume of clay (Vcl)^50^ is given below
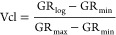
1where GR_log_ is the γ-ray reading of the formation, GR_min_ is the minimum γ ray (clean rock), and GR_max_ is the maximum γ ray (shale). Porosities are computed from all three porosity logs including density, neutron, and sonic. Data reliability of density and neutron logs is checked with the help of the density correction curve, as both the tools are sensitive to the borehole quality. The formula used for calculating the density porosity (φ*D*)^50^ is
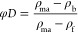
2where ρ_ma_ is the matrix density (2.71 g/cm^3^ for limestone), ρ_f_ is the fluid density (1.1 g/cm^3^), and ρ_b_ is the bulk density recorded using the tool. The value for the neutron porosity (φ*N*) is directly taken from the compensated neutron log (CNL). Sonic porosity (φ*S*)^50^ is calculated using the transit time computed using the sonic log using the formula
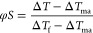
3where Δ*T*_ma_ is the matrix travel time (47.6 μs/ft for limestone), Δ*T*_f_ is the fluid travel time (185 μs/ft), and Δ*T* value is the sonic log response in the formation. Average porosity or total porosity (φ*T*)^50^ is the average of at least two different porosities among the density, sonic, and neutron porosities. Preferably, if the neutron and density log data are available, the total porosity is computed using these two logs.
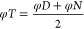
4To determine the amount of connected pores within the rock volume, effective porosity (φ*E*)^50^ is computed, which is the product of the total porosity and volume of clean (1 – *V*_sh_). This eliminates the non-connected pore space representing clay.
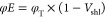
5The Sakesar Limestone is composed of clean massive, bedded limestone units; therefore, fluid saturation is computed with the help of Archie’s equation.^50^ The formula used for calculating water saturation is
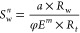
6where *S*_w_ is the water saturation, *R*_t_ is the deep resistively, *n* is the saturation exponent, *m* is the cementation factor, and *a* is the tortuosity factor.

### 3.2 Pore Pressure Prediction

Pore pressure in sedimentary formations is not always equal to hydrostatic pressure. It is more than the hydrostatic pressure (overpressure), and sometimes, it is double compared to the hydrostatic pressure. If abnormal pressure is not calculated properly, it might cause drilling hazards such as blowouts, kicks, etc. To prevent these events, it is important to predict the pore pressures.^51^ Pore pressure analysis of the Balkassar field is performed with the help of the sonic log-based Eaton’s method.^52^

Overpressure is mostly caused when the fluid in the pores is not expelled at a rate at which the sediments are deposited.^26^ Clays play a vital role in the generation of overpressure conditions because pore pressure is preserved in clay because of its zero permeability. Identification of clay-rich intervals is done by determining the clay intervals by drawing a clay baseline using the GR curve. A line group is manually drawn on the GR log curve, which is taken to be around 70 API to separate the clay points from the non-clay ones. Overburden pressure or the lithostatic pressure is the major reason for the fluid expulsion form the pores.^53,54^ Overburden pressure (OB) can be calculated using the density data. To predict the pore pressures, OB is required, which is a crucial step in this process since the pore pressure and fracture pressure are often calculated directly from the OB. The equation for calculating the OB^55^ is
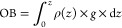
7where ρ is the density, *g* is the gravity acceleration, and *z* is the depth.

In normal geologic settings, hydrostatic pressure is the normal pressure or the pressure exerted by the column of water from the formation depth to the sea level. If the pore pressure of a formation at a given depth is greater than the hydrostatic pressure, the pressure is said to be overpressure.^56^ Whereas if at any given depth, the formation pressure is lesser than the hydrostatic pressure, the formation is considered underpressured. The hydrostatic pressure (HP) is calculated using the formula^24,57^
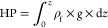
8One of the key components in pore pressure prediction is to define a normal compaction trend line. Under mechanical compaction, porosity reduction is linked with the expulsion of the fluid out of the pores in response to the overburden pressure. In the case of the slow sedimentation rate, fluids can be easily expelled out of the pore spaces to maintain equilibrium with the compaction rate. In this scenario, porosity is reduced at a predictable rate and the rock is said to be compacted under the normal compaction trend (NCT).^25^

The pressure exerted by the fluid within the pore space of a porous formation is termed as pore pressure. Terzaghi’s and Biot’s effective stress law provides the basis for the pore pressure prediction.^58,59^ According to this theory, the fluid pore pressure is a function of the total stress and vertical effective stress. There are several methods proposed for predicting the pore pressure using interval velocity and transit time from well log data. Eaton’s method is one of the more widely used quantitative methods. Eaton (1975)^52^ provided an empirical relation for pore pressure prediction using the sonic transit time of the compressional wave
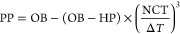
9where PP is the pore pressure (psi) and NCT is the normal compaction trend line. The exponent value represents Eaton’s index, which must be selected by calibrating the predicted pressure with the measured pressure data available at various depths within the Sakesar Limestone. Fracture pressure (FP) is the pressure required to fracture the formation and cause mud loss from the wellbore into the induced fracture. FP is a point where the formation breaks,^60^ and Eaton’s Method is used for its calculation. The following equation is used in the calculation
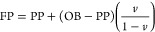
10where FP is the fracture pressure and *v* is Poisson’s Ratio (dimensionless).

### 3.3 Geohistory Analysis

Geohistory analysis is used for determining the burial history (subsidence and uplift) and its related processes controlling the compaction of sediments.^53^ Burial history represents all the changes with respect to the compaction and heat flow. After generating these burial history plots, the porosity reduction rate and pore pressure are computed for the Tertiary rocks. An absolute time scale must be used to define the events (such as deposits, hiatuses, or unconformities) chronostratigraphically. In this study, a geological time scale found elsewhere^61^ is used for this purpose. The necessary input data used for generating the burial history include the generalized stratigraphy, formation thickness, formation depths, deposition ages, lithologies, and petroleum system information (Table 2).

**Table 2:** Input data used for creating the burial history plot of the Balkassar OXY 01 well. A total of nine events up to the Paleocene age formations have been used to prepare the burial plot, for which information of the event end age, total depth, encountered thickness, petroleum system component, and lithology is utilized

S#	event name	event type	end age (my)	top depth (m)	present thickness (m)	petroleum system	lithology
1	Nagri	formation	9	0	478		sandstone
2	Chinji	formation	14	478	930		clay
3	Kamlial	formation	16	1408	106		sandstone
4	Murree	Formation	20	1514	907	seal	sandstone
5	Oligocene	Hiatus	25				
6	Chorgali	formation	35	2421	46	reservoir	limestone (Shaly)
7	Sakesar	formation	41	2467	135	reservoir	limestone
8	Patala	formation	58	2602	22	source	shale
9	Lockhart	formation	62	2624	35		limestone (organic rich)
10	Hangu	formation	66	2659	27		sandstone

The burial history plot is generated till the Paleocene age, for which a total of 10 stratigraphic events have been used as the input data, including 9 formations and 1 hiatus (Oligocene age). Thickness of each formation is then calculated using the formation tops, and lithologies are assigned to each formation. The burial history plot is generated in Basin Mod software, which creates compaction and porosity relationship using porosity–effective stress compaction to compute the porosity reduction rate with burial depth. The fluid flow model explains that the porosity of rocks is controlled by the sediment compaction, which is a function of pressure.^62^ Using the computed porosity from the log data, the porosity reduction rate is computed for the Balkassar OXY 01 well.

### 3.4 Subsurface Structural Interpretation

**Figure 4:**
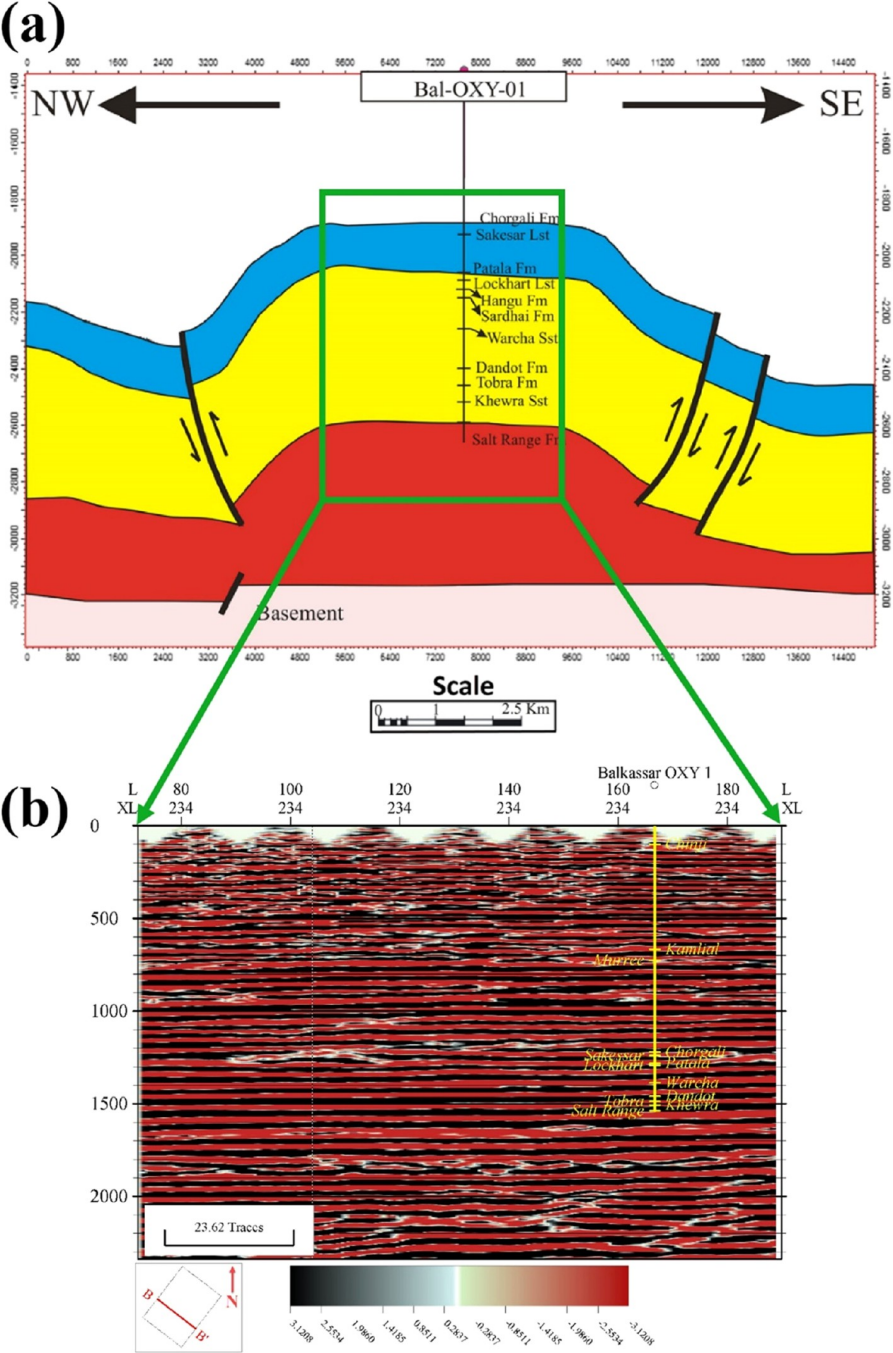
Model of the Balkassar anticline (a) bounded by faults on its northwest and southeast flanks and (b) uninterpreted seismic section of crossline 234. In (a), the Balkassar anticline represents a broad flat box fold geometry drilled up to the Precambrian Salt Range Formation. Fold limbs are marked by a major fault in the northwest and two faults in the southeastern side. The basement is also displaced by a normal fault. In (b), the crossline covers the central flat part of the Balkassar anticline as represented in (a), depicting flat smooth horizons throughout its lateral extent. The well pick of Balkassar OXY 01 along with the drilled formations is shown in yellow color (modified from ref (40)).

3D seismic data of the Balkassar area are used for this study, but as per public domain data limitation, only a 10 km^2^ seismic cube is available. The current data set only covers the central flat part of the broad box fold and does not cover the flanks of the Balkassar anticline (Figure 4a). Crosslines show flat reflectors in the seismic section, which are most suitable for determining the effect of compaction on the porosity and pressure. The uninterpreted seismic section of crossline 234 is shown in Figure 4b, depicting the flat reflectors covering the central part of the Balkassar anticline.

The well-to-seismic tie is established with the help of the check shot data of the Balkassar OXY 01 well for marking three horizons including Chorgali, Sakesar, and Lockhart formations. Time of the Chorgali Formation at the Balkassar OXY 01 well location is 1.32 s. The Sakesar Limestone is marked at 1.36 s, and the Lockhart Limestone reflector is marked at 1.4 s. There are no prominent faults in the study area, as the data are only covering the flat central part the structure. The depth structure map of the Sakesar Limestone is generated using the time structure map and velocity information for determining the structural relief of the Sakesar horizon in the subsurface.

### 3.5 Seismic Porosity Estimation

SI is a particularly important process in determining hydrocarbons and reservoir characterization. Seismic data contain information about the interface between two layers, which can be converted into layer information with the application of SI. This can then be correlated with well data, thus helping in reservoir characterization. SI is a reverse process, and it is known as “inverse modeling”. The seismic trace, which is the result of convolution of the reflection coefficient with the wavelet, is used in this process to find the reflection coefficient. This is done by multiplying the seismic trace with an inverse wavelet, which is extracted from the seismic data.^63^

Model-based seismic inversion (MBSI) is applied on the Balkassar 3D seismic cube. A zero-phase seismic wavelet of 200 ms and 18 HZ frequency is extracted from the cube to convert seismic reflection into acoustic impedance. After extracting the seismic wavelet, seismic data are correlated with the synthetic trace produced at the well location.^64^ The RMS error and correlation coefficient are calculated, and 82% correlation value is achieved. Seismic data are band-limited with no low and high frequencies, which are lost during convolution.^65^ To obtain the absolute value of impedance, low frequencies ranging from 0 to 16 HZ are added using MBSI and a low-frequency model is generated. Once low frequencies are added into the data, a P-impedance model is generated using density and sonic logs.

The most difficult parameters in the reservoir characterization are the prediction of porosity and permeability.^66−68^ This is because of the fact that these two parameters may vary significantly within the reservoir.^69^ Machine learning and ANNs have become a focus of interest in the modeling of complex rock systems in recent years.^70,71^ The probabilistic neural network (PNN) technique is used for the computation of porosity from seismic data. The computed porosity from the Balkassar OXY 01 well is inverted using the log porosity with the seismic cube and inverted P-impedance model. The inverted porosity curve is trained with the log porosity using multiple seismic attributes to achieve the best correlation. This process is performed to build a relation between the log and seismic data. After achieving the best correlation of the established relation, PNN applies it on the whole seismic cube to predict the lateral and vertical variations of porosity.

## 4 Results

### 4.1 Quick-Look Petrophysics

**Figure 5:**
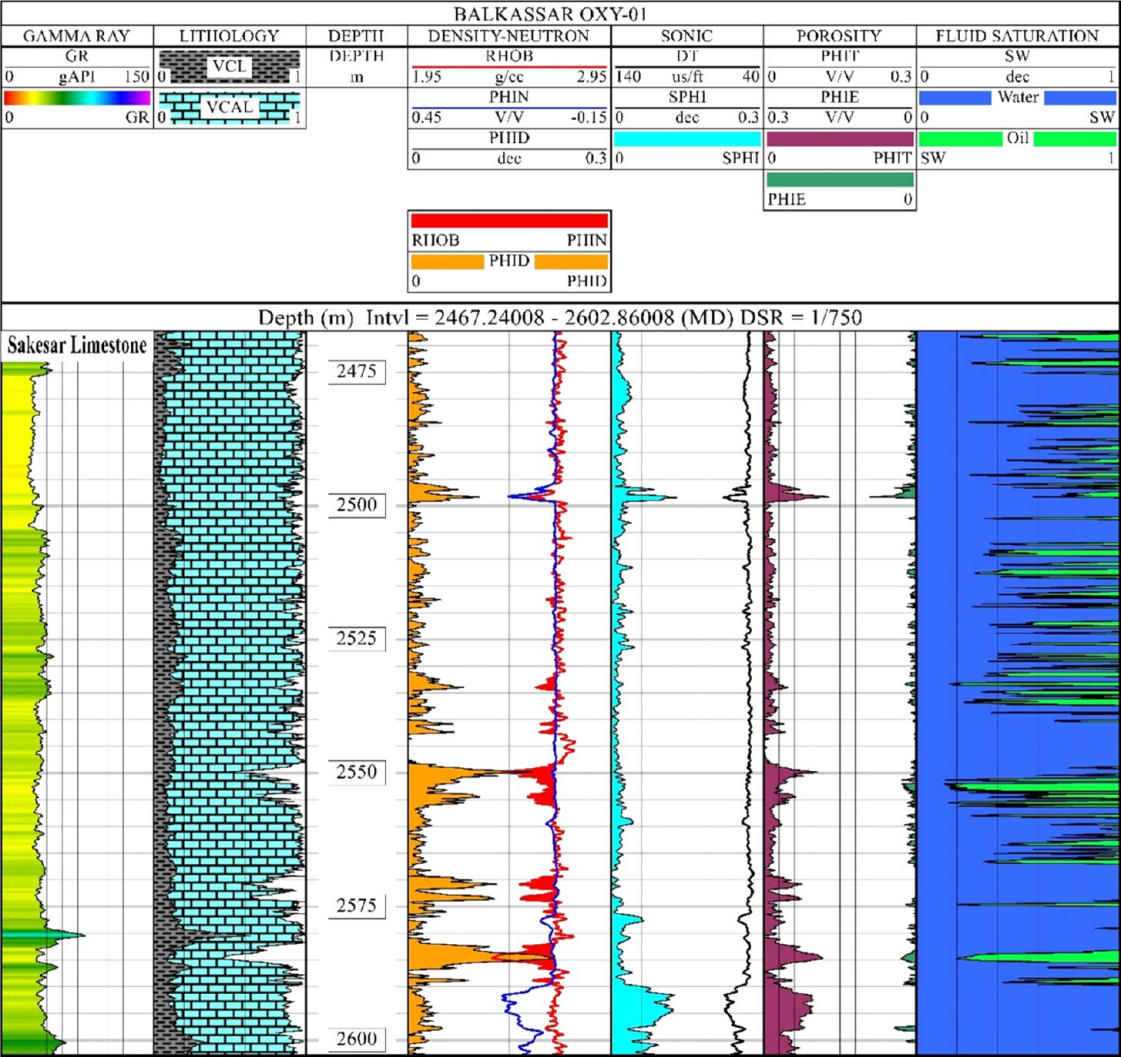
Wireline log interpretation of the Balkassar OXY 01 well showing conventional formation properties computed for the Sakesar Limestone. A total of six tracks are shown to display the computed rock properties including volume of clay, porosities, and fluid saturation. The volume of clay (gray) and volume of calcite (blue) are shown in the lithology track, representing dominant the carbonate facies of the Sakesar Formation. Density porosity (mustard), sonic porosity (cyan), total porosity (purple), and effective porosities (green) are calculated to determine the porosity variations with respect to depth. The effective porosity curve depicts tight formation conditions within the Sakesar Limestone. The fluid saturation track represents that the formation is mostly water-wet (blue) with few intervals of oil-bearing zones having less thickness.

Complete analysis of the quick-look petrophysical interpretation for the Sakesar Limestone in the Balkassar OXY 01 well is shown in Figure 5. A total of six (6) tracks are used for displaying the results of reservoir properties, and the data set is plotted against depth in metric units. Raw log curves containing GR, RHOB, PHIN, and DT are displayed, whereas the computed results include lithology, porosities, and fluid saturation curves. Standard scales of the curves are used for raw log curves. Lithology and fluid saturation are plotted with a range of 0–1, and the computed porosities of all the curves are plotted in fractions using a scale of 0–0.3.

The GR log curve is displayed in the γ-ray track with the color fill range to show the variation of log curve values. The Sakesar Limestone is composed of massive limestones, which represents a smooth γ-ray curve with low values. This smooth and consistent log curve trend of GR log represents massive bedding of carbonate units within the Sakesar Limestone. A few small peaks of relatively high GR values are observed at the bottom of the formation. Using the multimineral model based on the GR and porosity data, lithological facies are computed, which represent a high percentage of the Limestone. It can be clearly observed that the formation comprises more than 85% of calcite minerals with minor clay facies.

The density–neutron track displays RHOB and PHIN curves, and in most of the interval, both the curves overlie each other with minor or negligible separation. This upper part of the formation represents very tight conditions in terms of porosity. Within the lower half of the formation below the depth of 2550 m, few zones are marked by neutron–density cross-over, indicating probable hydrocarbon-bearing zones. There are few fluctuations in the density curve, which indicate few good porosity intervals, as depicted by the density porosity (PHID) curve plotted.

The sonic track includes the DT log curve, which also shows a smooth and consistent behavior apart from few peaks, indicating possible fractured zones. The lowermost part of the formation at the depth of around 2590 m indicates a highly porous zone, which can be observed from the sonic porosity (PHIS) curve. This high-porosity zone is also marked by the PHIN curve, but PHID does not show an appreciable increase, as RHOB values are relatively higher in this interval. This is the reason why relying on a single log result could be misleading while interpreting petrophysical properties, as all three porosity logs are studies on different principles. This limitation of porosity logs is catered by obtaining the average of at least two curves to get a better representation of porosities. In this regard, the total porosity (PHIT) is computed by taking the average of already computed porosity curves (preferably PHID and PHIN). Average values of PHIT in the top interval of the formation are less than 2%, whereas they increased in the lower part, ranging between 3 and 4%.

Connectivity of the pores is highly necessary for the fluid flow, which can be determined by computing the effective porosity (PHIE). The computed PHIE curve, plotted in the porosity track, confirms the presence of tight formation conditions for the fluid flow, as the values are extremely low, averaging around 1–2%. However, this little porosity present in the formation is mostly saturated with water, represented by a high value of water saturation (Sw) reaching up to 90%. There are only few peaks of hydrocarbon saturation (Sh) shown in the fluid saturation track.

### 4.2 Pore Pressure Prediction

**Figure 6:**
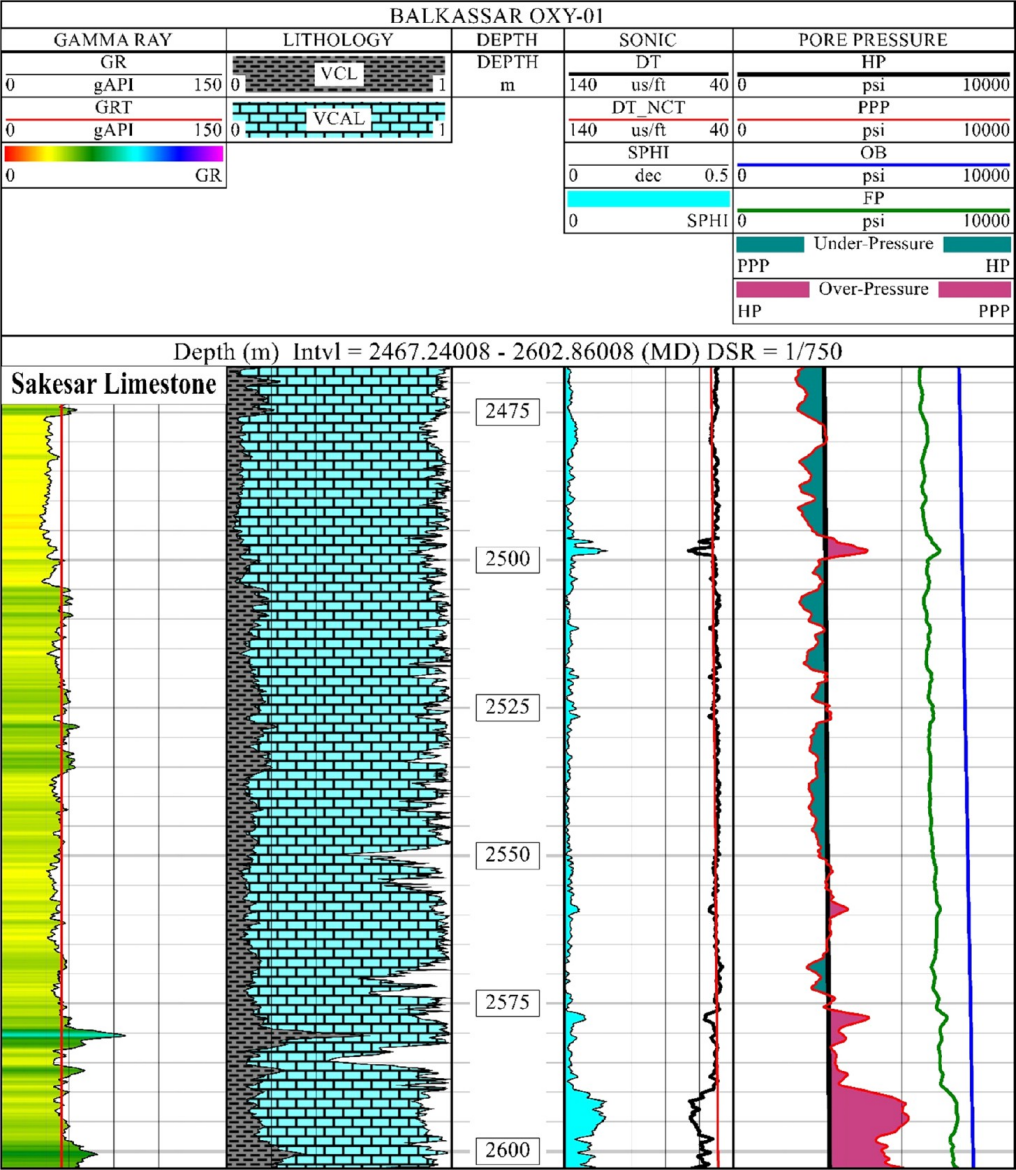
Pore pressure analysis of the Sakesar Limestone in the Balkassar OXY 01 well. Lithology, porosity, and pressure are related to determine the reservoir properties of the formation. The γ-ray curve is plotted in the first track, with volume of clay and calcite in the lithology track, the sonic porosity and normal compaction trend in the sonic track, and fluid pressures in the pore pressure track. A high percentage of calcite with low porosities in most of the formation thickness indicates the normal compaction trend (NCT) with underpressure (green color fill) conditions except for the lowermost 10 m interval. This high-porosity zone at the lowermost part shows overpressuring (purple color fill), which is also indicated by the deviation of the sonic curve from the NCT. The hydrostatic pressure (HP) curve is plotted in black color, with overburden pressure in blue color and fracture pressure in green color.

Pore pressure prediction for the Sakesar Limestone is performed using Eaton’s method for determining the fluid pressure conditions within the formation. Detailed analysis of the PP results is given in Figure 6. A total of four tracks are used for displaying the results of PP analysis. Tracks 1 and 2 show the lithological distribution as discussed in the quick-look petrophysical interpretation. The γ-ray trend line (GRT) is marked with a cutoff value of 70 API for defining the high clay points. These clay points depict the intervals having more clay percentage lithology with minimum or negligible permeability. The DT curve is used for defining the normal compaction trend (DT_NCT), and both the curves are displayed in track 3 using the same scale. The DT_NCT curve displays a decreasing trend of sonic values, and this typical trend indicates an increase in compaction with burial depth. At the base of the Sakesar Limestone at around the depth of 2590 m, the DT curve deviates from the normal compaction trend line, marking the top of the overpressured zone.

The pore pressure track displays the computed results of pressure curves including HP, OB, PP, and FP curves with the same scale of 0–10,000 psi. Except for only the 3 m interval at a depth of around 2497 m, the Sakesar Limestone exhibits underpressure conditions till the depth of 2575 m. This 3 m interval possibly represents a fractured zone, as indicated by the sonic peak deviating from the normal compaction trend. Overall low porosities depicting tight conditions compliment these underpressure zones. Below the depth of 2575 m, an increase in the PP is observed, which abruptly increases at the depth of 2590 m, indicating a overpressure zone. This 10 m interval of the formation shows difficult drilling conditions, as the difference between the PP and FP curves decreases drastically. Apart from this, the Sakesar Limestone bears safe and stable drilling conditions, as FP values are considerably higher than the PP values.

### 4.3 Geohistory Analysis

**Figure 7:**
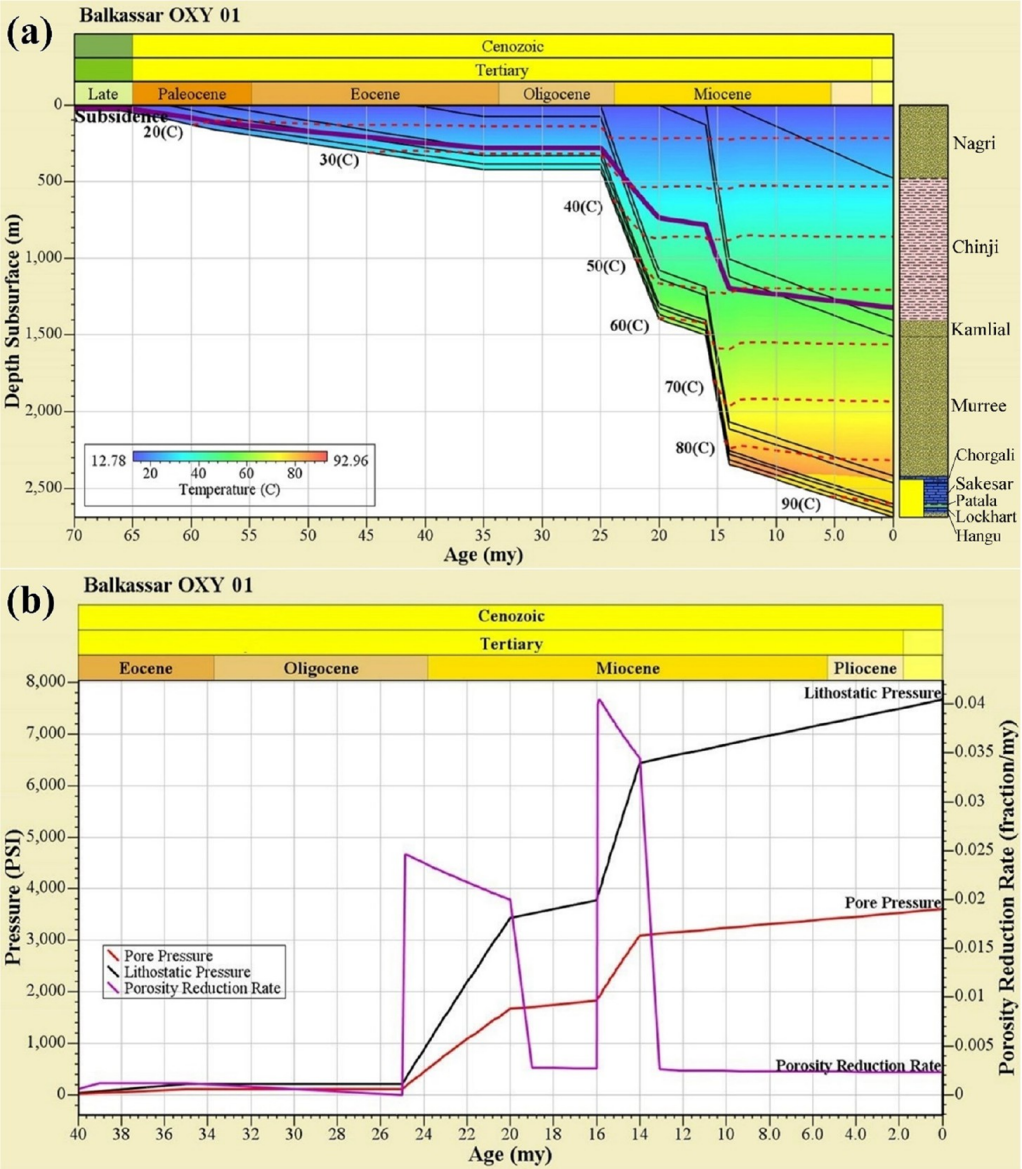
(a) Burial history plot for the Cenozoic Era of the Balkassar OXY 01 showing subsidence curve (purple) and isotherm lines (red dash lines) along with the burial depth. The plot represents two major subsidence episodes during the Miocene times, indicating a high sedimentation influx resulting in increased overburden pressure. Isothermal lines depict that the Sakesar Limestone achieved a temperature of up to 90 °C at the depth of around 2600 m. Lithological formations drilled in the well are shown in the form of a litholog at the right vertical axis. (b) Porosity and pressure plot of the Sakesar Limestone in the Balkassar OXY 01 showing the relationship of pore pressure and porosity reduction rate with an increase in lithostatic pressure. Two major events marked by high porosity reduction rates are observed during the Miocene time, which are the result of the subsidence events marked in Figure 8. These high subsidence rates reduced the formation porosities between 25–19 and 16–13 my.

The burial history of the Balkassar OXY 01 well using the stratigraphic detail is generated to understand the sedimentation and subsidence rate with burial depth. The focus of this study is to determine the effect of compaction on the Eocene reservoir in the Balkassar area. The Balkassar OXY 01 well is used to generate the burial history plot for evaluating the target formation, Sakesar Limestone (Figure 7a). The depositional sequence of the Eocene limestone shows a gradual increase in depth till the end of the Oligocene age. With the increase in the overburden thickness, around 25–20 my, the subsidence curve is also showing a drop of about 500 m. However, during the Early Miocene from 20 to 16 my, a relatively flat subsidence curve can be observed in this burial history plot. With the start of the deposition of the Chinji Formation, an abrupt subsidence event can be observed at 16 my, which might affect the porosities and pressure of the formations buried underneath. Temperatures plotted on the burial history plot range from 0 to 90 °C.

The pore pressure plot in response of the porosity reduction rate is computed for the Sakesar Limestone to determine the relationship between these two parameters (Figure 7b). The purple curve shows the porosity reduction rate (fraction/my) with time, the red curve represents the PP curve (psi), and the black curve indicates the lithostatic pressure or OB. Flat curves of pore pressure and porosity reduction rate till Oligocene times are observed, as the Oligocene age is represented by a nondepositional unconformity. It can be seen that there are two episodic events, which mark a sudden increase in the porosity reduction rate, i.e., 25–20 and 16–14 my (Figure 7b). These two time spans are indicated by a high subsidence rate identified in the burial history plot (Figure 7a). This is caused by the post-Eocene collisional phenomena followed by the high sedimentation influx of molasse sediments during the Miocene age.

### 4.4 Subsurface Structural Interpretation

**Figure 8:**
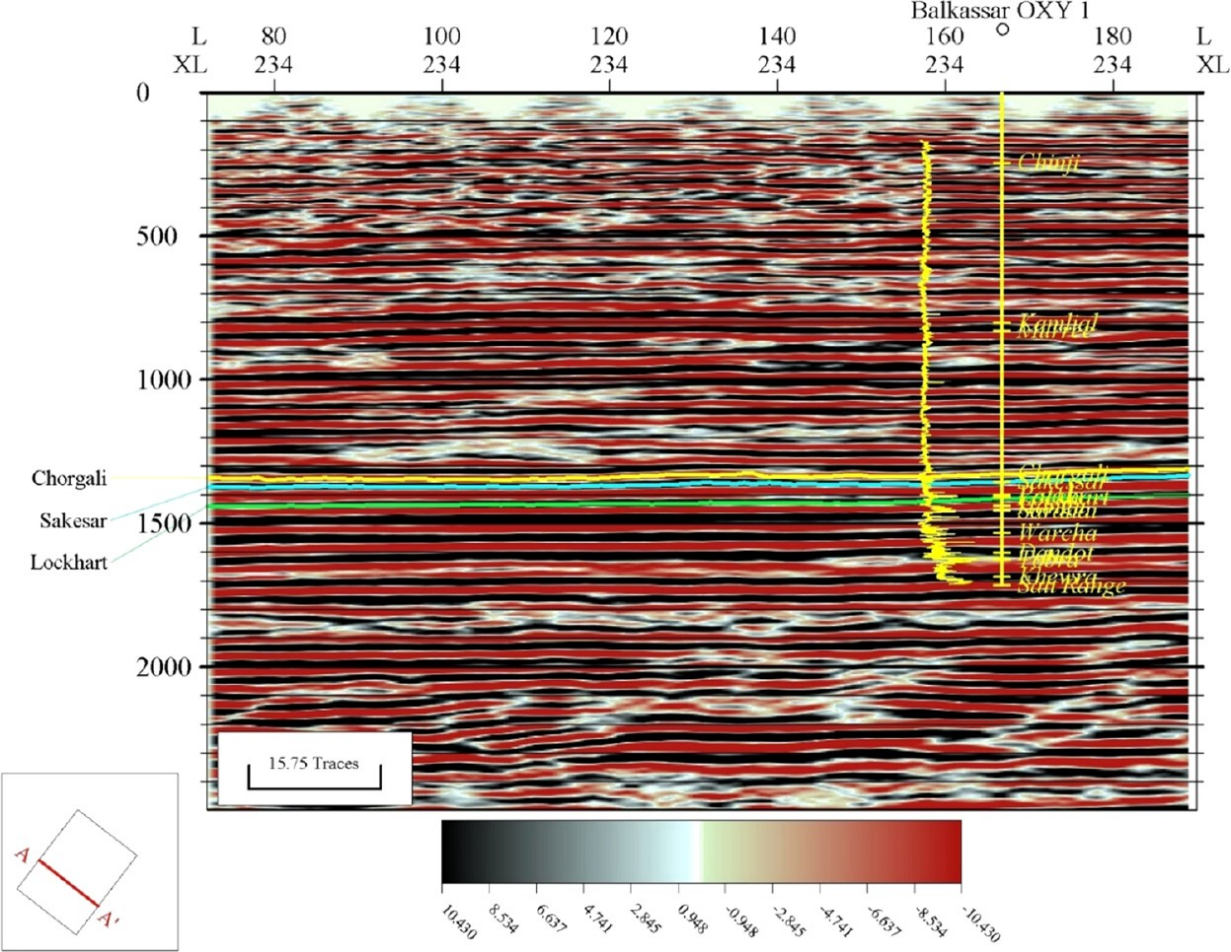
Interpreted seismic section of crossline 234 at the Balkassar OXY 01 well. Three horizons including the Chorgali Formation (yellow), Sakesar Limestone (blue), and Lockhart Formation (green) are marked on the seismic section. The well Balkassar OXY 01 along with formation picks is plotted on the section. The horizons show a flat trend, as the seismic cube only covers the central flat portion of the Balkassar box fold anticline.

**Figure 9:**
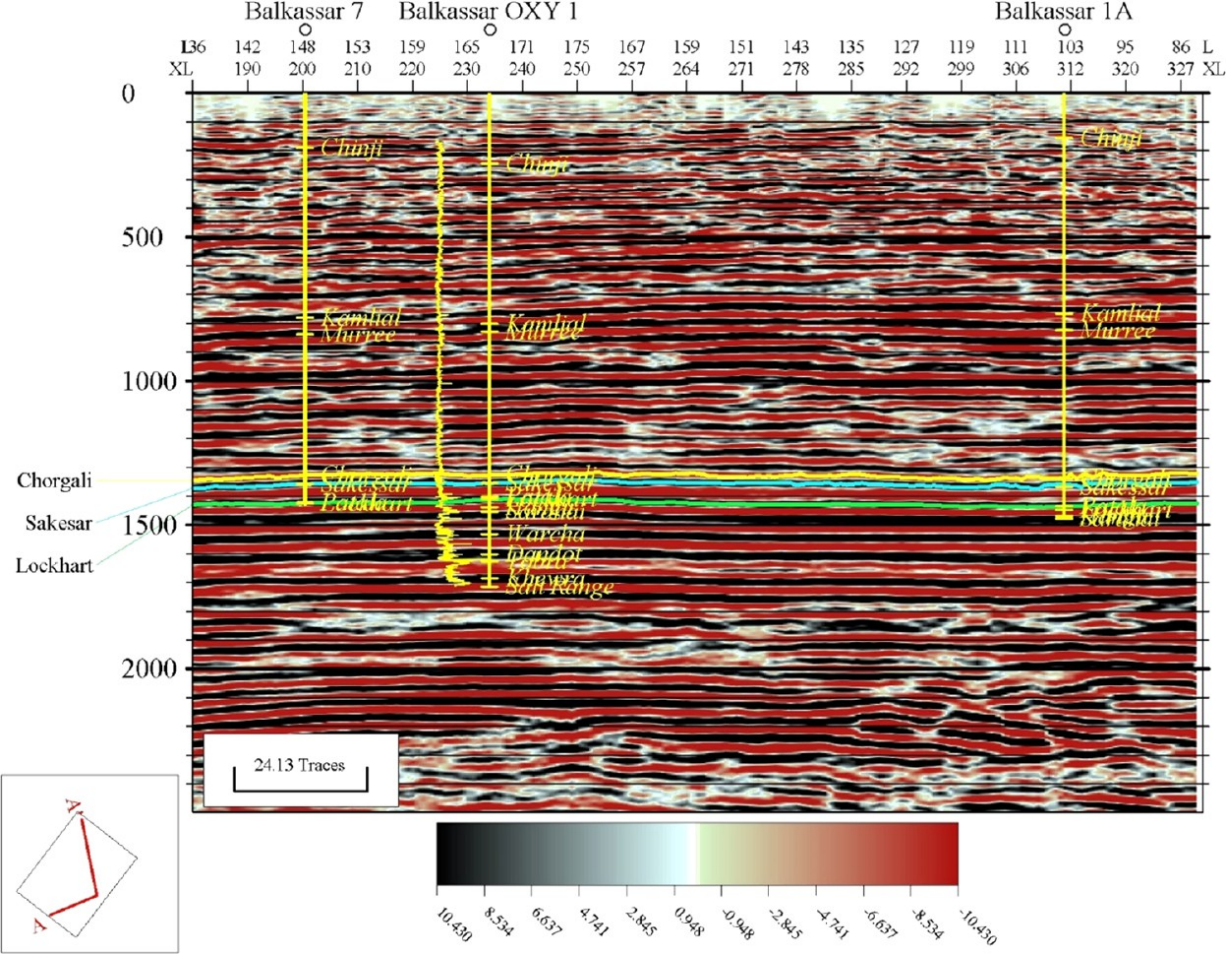
Interpreted arbitrary seismic line passing through all three wells of the study area. The line is selected to pass through all three wells used in the study to show the regional trend in the north–south direction. A gentle bulge is observed toward the Balkassar 07 well in the southern part of the study area. Formation picks of all drilled formations in three wells are shown in the figure with yellow color.

The Balkassar structure is represented by a broad box-shaped anticline bounded by reverse faults in the southeast and northwest directions. The interpreted seismic sections of crossline 234 at the Balkassar OXY 01 well location are displayed in Figure 8, which cover the central flat part of this box fold. The well bore is plotted along with the displayed GR curve on the left side of the well bore, and the formation tops are also displayed on the seismic section. The seismic-to-well tie is established using the check shot data of the Balkassar OXY 01 well. The orientation of the crossline is in the NW–SE direction. Overall, all the reflectors are lying horizontally, but there is a very slight dipping trend of some milliseconds observed in the NW direction. An arbitrary line, generated passing through all three wells, is shown in Figure 9. This line is initiated from the Balkassar 01A well to the Balkassar OXY 01 well in the north–south direction and continues toward the Balkassar 07 well drilled in the southwest. The arbitrary line shows the lateral continuity of the strata in the subsurface.

**Figure 10:**
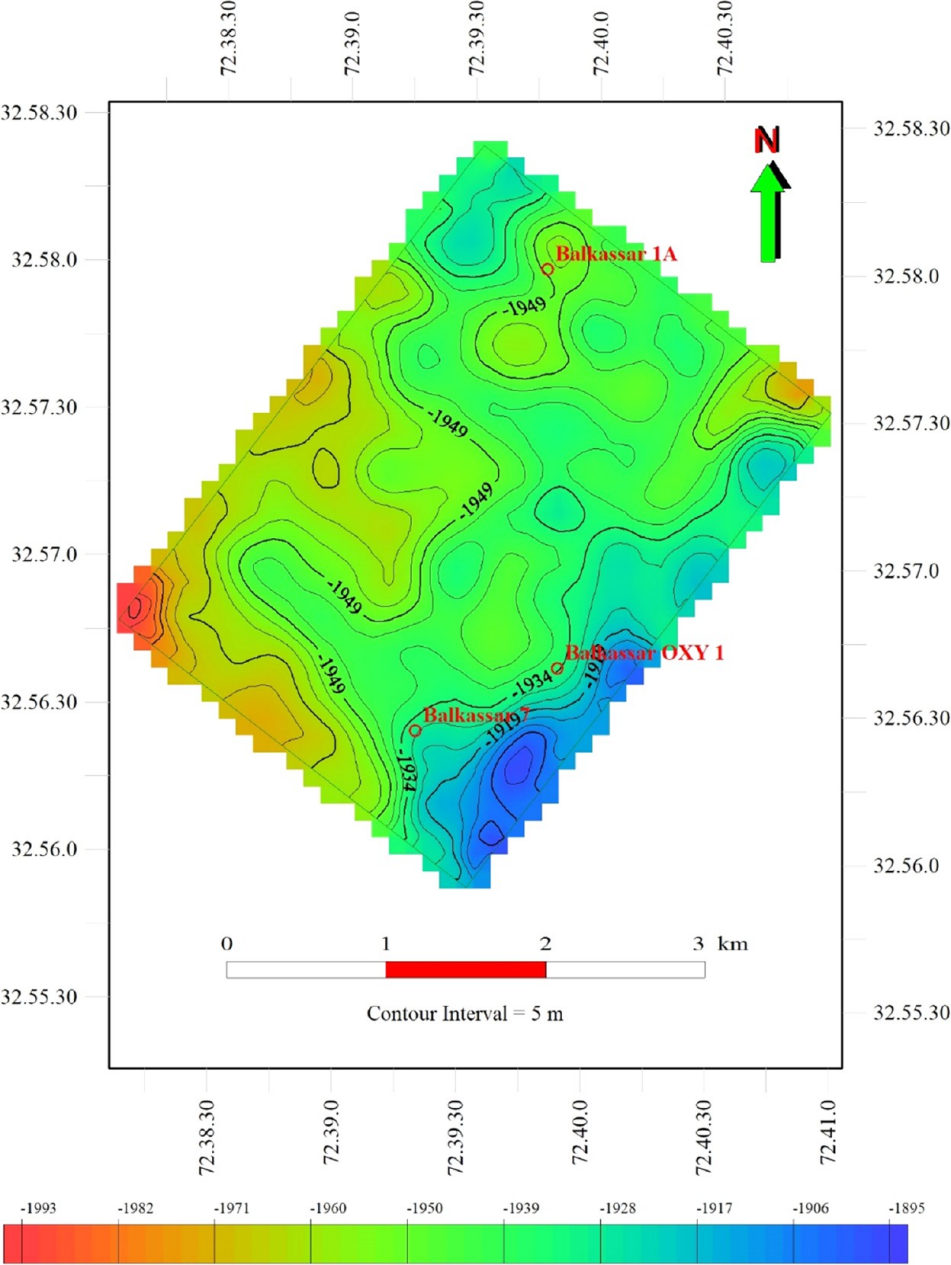
Depth structure map of the Sakesar Limestone in the Balkassar field. Contour values along with the color fill are used to display the depth variations of the marked Sakesar horizon. Blue color represents the shallow part in the southern part of the study area, and red color shows the deeper portion in the western corner. Most of the area in the depth map is represented by the green color fill, as the seismic cube covers only the central flat part of the anticline. Subsea depths of the formation are plotted in the map.

The depth structure map of the Sakesar Limestone is generated with a 5 m contour interval, using two-way time and velocity maps as input data (Figure 10). Color fills show the increase and decrease in the time values. The blue color fill shows less time values indicating a shallow structure, whereas the red color shows the deeper level in all the maps. Overall, 80% of the depth structure map shows a green-colored filled zone showing a relatively flat depth surface. A small red color fill zone in the western part of the study area and the southeastern part of the study area shows a shallower structure. This southeastern shallower part of the structure has been the target for drilling wells in search of hydrocarbons.

### 4.5 Seismic Porosity Estimation

**Figure 11:**
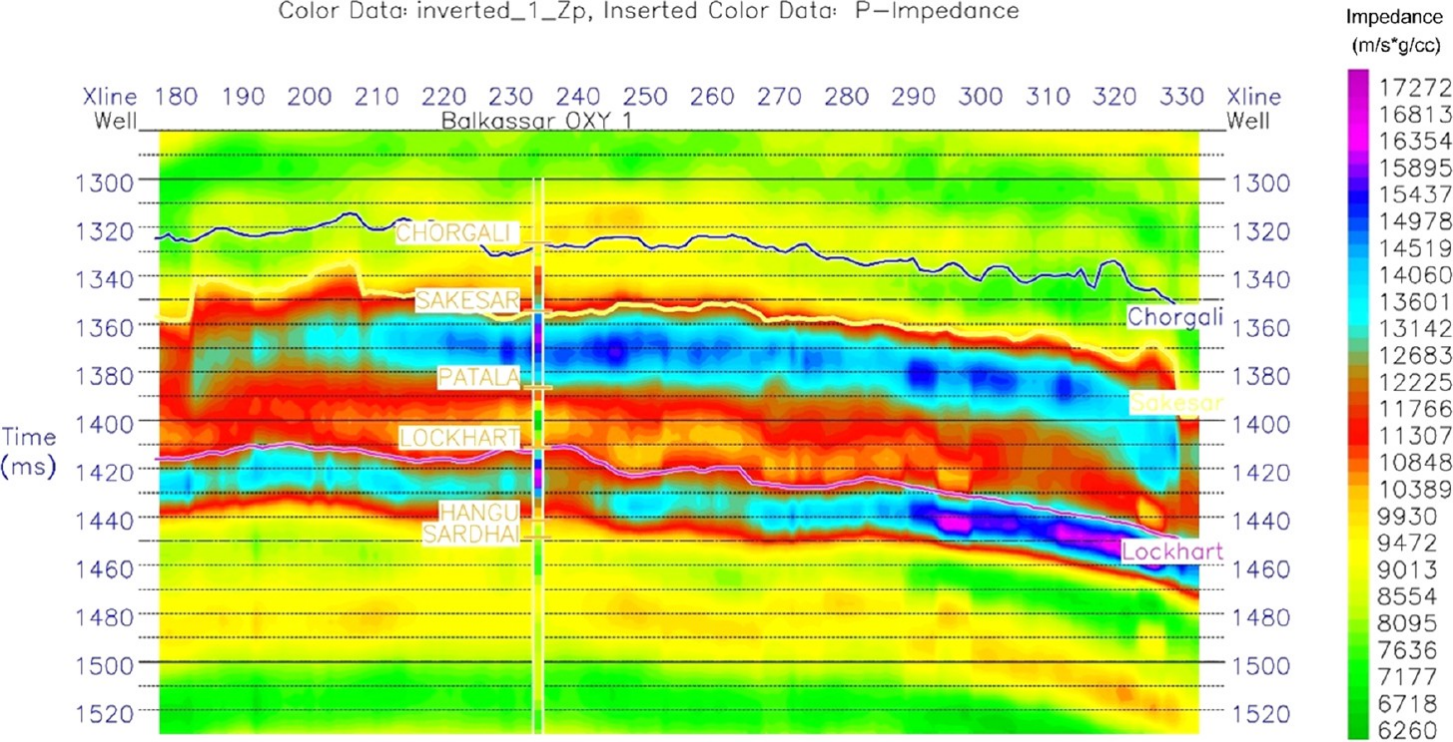
Inverted P-impedance section of the study area through the Balkassar OXY 01 well using MBSI. The section covers all the marked horizons of Chorgali, Sakesar, and Lockhart formations along with other encountered formations. The color fill is used to represent the impedance variations with green color representing the low impedance values, and magenta color shows the high impedance. The Sakesar Limestone shows high impedance values, shown by the blue color fill, depicting tight formation conditions. However, a relatively low-impedance zone within the Sakesar is marked in the northwest of the section.

Results obtained from MBSI are of excellent quality and in high resolution. An inverted P-impedance model of inline 167 (NE–SW) generated at the well location of Balkassar OXY 01 is shown in Figure 11. P-impedance values of the Sakesar Limestone range between 11,307 and 15,437 (m/s*g/cm^3^) as per the color bar distribution of impedance values. Top of the formation is marked by a low-impedance layer of about 11307 (m/s*g/cm^3^) throughout the seismic line. The middle part of the formation is characterized by high impedance values ranging up to 15,437 (m/s*g/cm^3^). There are not much lateral variations of impedance observed within the Sakesar Limestone, and most of the interval is represented in light blue with some dark-blue patches of high impedance. However, in the southern part, there are few low-impedance zones shown by reddish green color.

**Figure 12:**
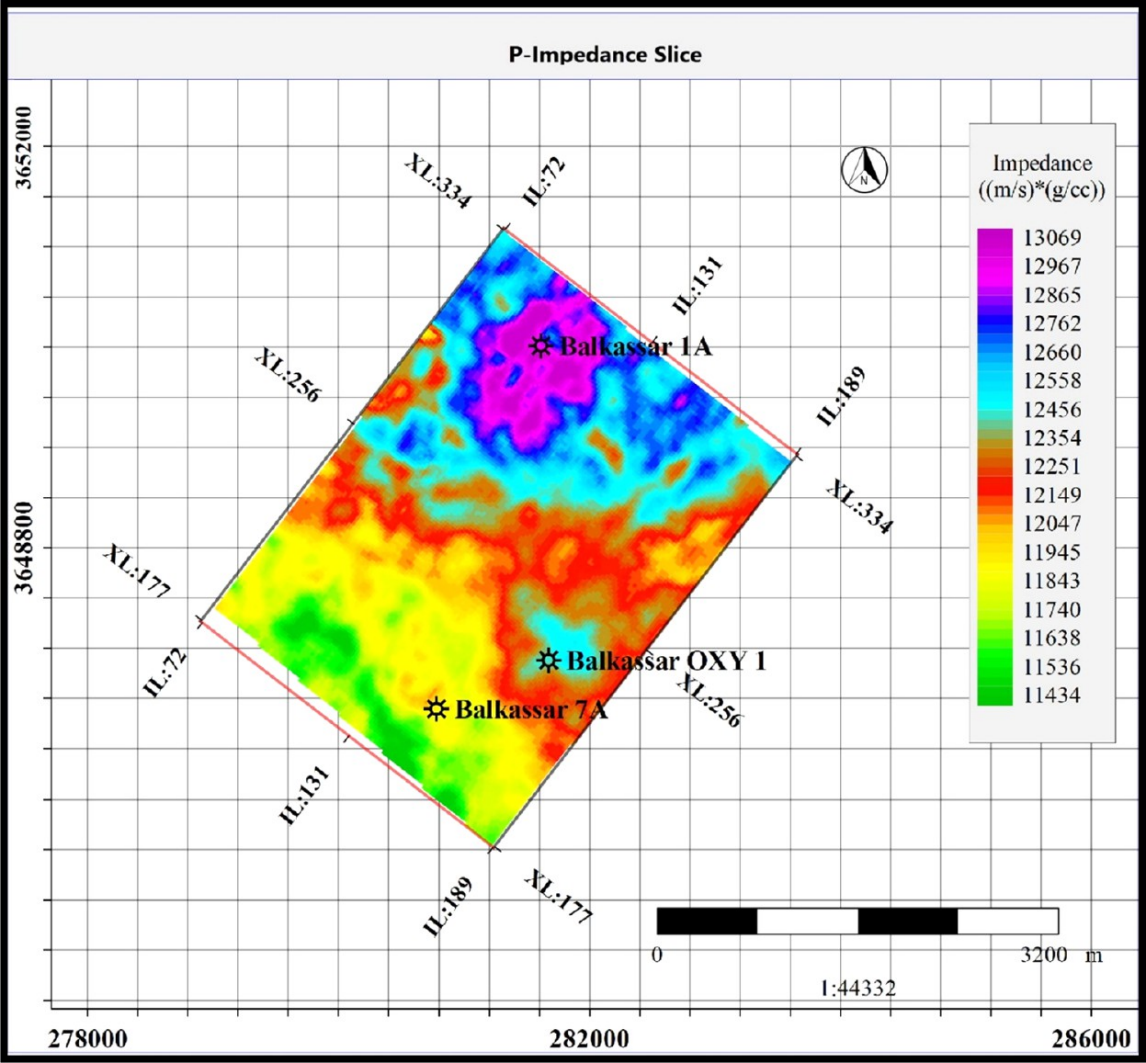
Inverted Zp slice extracted from the P-impedance model at the top of the Sakesar Limestone. The impedance slice of 20 ms averaging window is selected to display the lateral variations of impedance throughout the area with the help of color fills. The northern part of the area where the Balkassar 01A well is drilled represents a high-impedance zone (magenta color fill), whereas the southern part depicts low impedance values (green color fill).

The P-impedance slice extracted for the Sakesar horizon with 20 ms averaging window is shown in Figure 12. The P-impedance value of the Sakesar ranges between 11434 and 13069 (m/s*g/cm^3^). The slice of Sakesar represents a low-impedance zone in the southern part of the study area near the Balkassar OXY 01 and Balkassar 07 wells. These low-impedance zones are shown in green and yellow colors. High impedance values are observed around the Balkassar 01A well in the northern part of the study area marked by a purple color fill with a maximum value of 13,000 (m/s*g/cm^3^). The P-impedance range is a bit higher in the massive limestone units of Sakesar, and the petrophysical result also indicates extremely low porosities having tight conditions.

**Figure 13:**
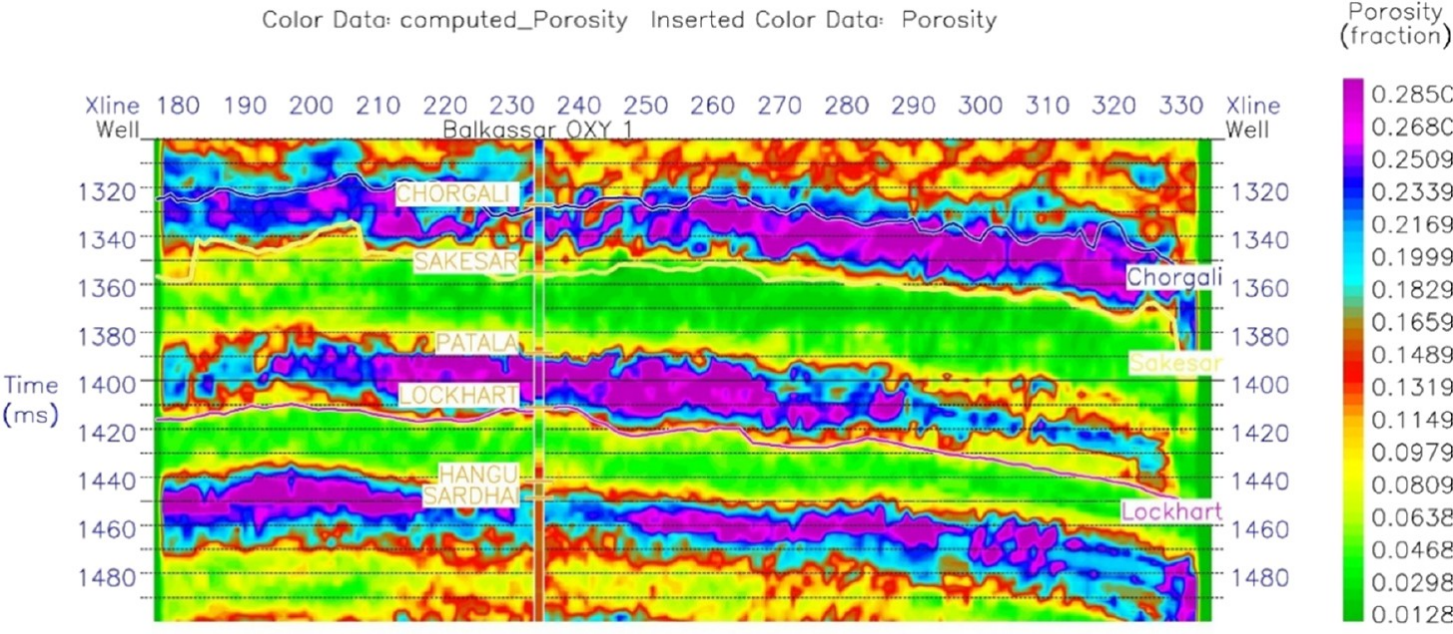
Computed porosity section of the study area through the Balkassar OXY 01 well using PNN. Predicted porosities in the Sakesar Limestone are less than 4% throughout the porosity section represented by the green color fill. This low porosity correlates with the porosity values computed from log data and validates the high impedance intervals.

**Figure 14:**
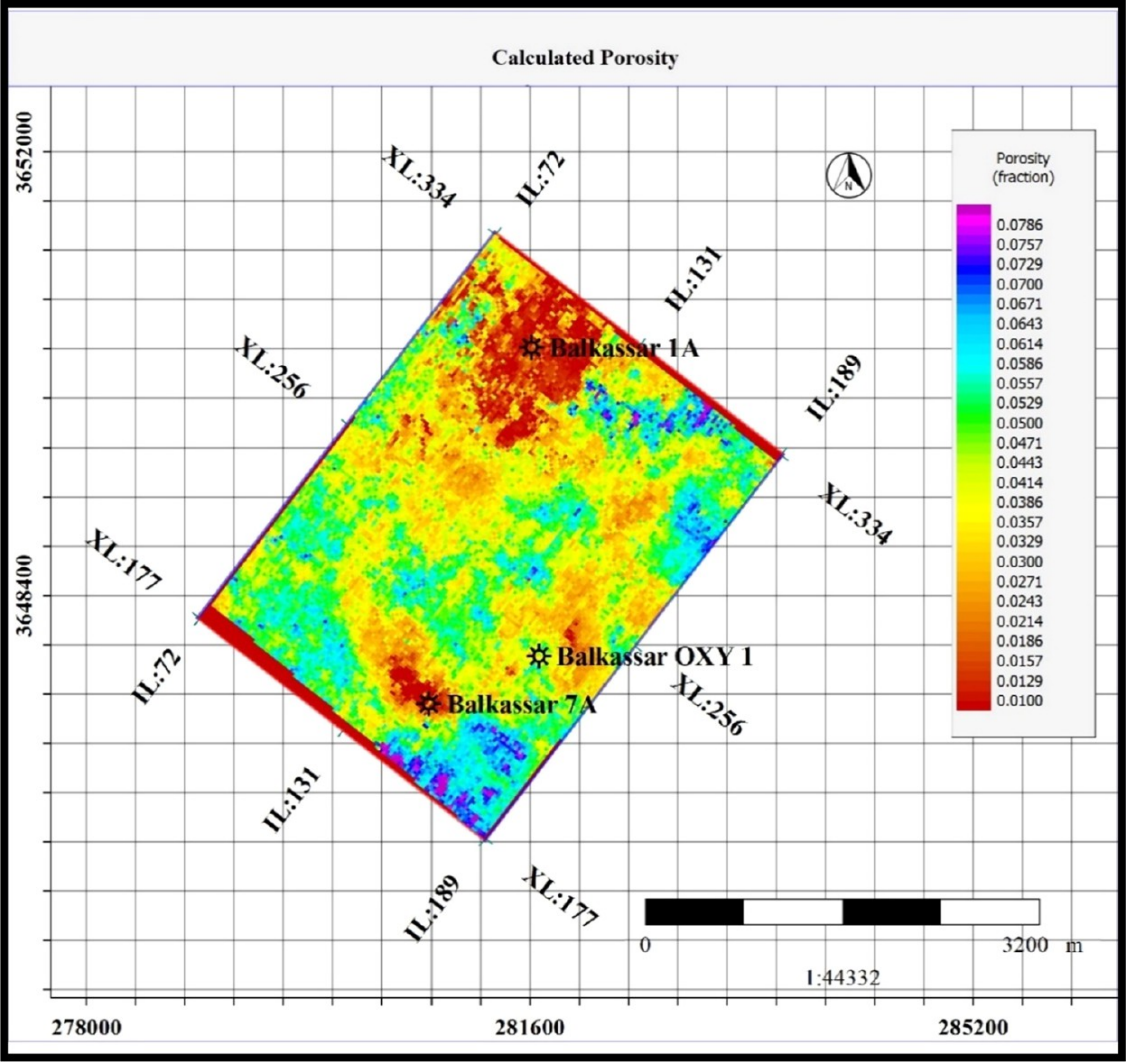
Porosity slice extracted from the computed porosity model at the top of the Sakesar Limestone. Predicted porosities using the PNN analysis show that the formation bears tight reservoir conditions throughout the study area. The northern part of the field represents extremely tight conditions with porosities less than 1%; however, the southern part of the study area exhibits a porosity range at 4–7%.

PNN is applied to predict the porosity over the whole impedance volume (Figure 13). Inverted porosity sections show good correlation with log-based porosity curves. Relatively less porosity values within the high-impedance zones justify the tight formation conditions. The color bar represented in dark green to light green colors represented the total thickness of the Sakesar limestone. Spatial porosity variation shows a slight increase in values toward the southern side of the well. A small zone of high porosity values of about 6–8% can be observed in the southern end of the inline 167, which is the same zone marked by low impedance values in Figure 11. These predicted porosity values show good correlation with the calculated total/average porosity of the Sakesar Formation through petrophysical analysis ranging between 2 and 4%. The porosity slice is then extracted from the computed porosity cube at the top of the Sakesar Limestone for displaying the lateral variations of porosities within the study area (Figure 14).

## 5 Discussion

Rapid sedimentation in younger basins greatly affects the reservoir quality of carbonates due to the high rate of subsidence. Compaction of sediments during burial involves the decrease of the bulk rock volume followed by the progressive decrease of porosity with increasing depth.^5,72^ Compaction is divided into two regimes, that is, mechanical and chemical compactions. The cause for the porosity reduction specifically due to compaction can be due to either mechanical processes that resulted in response to increased vertical effective stress^73^ or chemical ones that could be caused due to the dissolution and precipitation of different minerals.^74^ Porosity loss can be used to estimate how much sediments are compacted since deposition. Mechanical compaction being the primary mechanism of compaction^6^ is controlled by the overburden stress and subsidence. However, it is expected that mechanical and chemical compactions will always work together.

In siliceous sediments, mechanical and chemical compactions are rather well separated along with the burial depth. Mechanical compaction dominates at shallow depth, while chemical compaction becomes predominant at a depth of around 2–3 km corresponding to 60–80 °C. In carbonate sediments, these two effects are difficult to separate,^75^ as both the compaction processes occur simultaneously. However, the rate of mechanical compaction at the shallow depth is extremely high in carbonates.^9^ On the other hand, within clastic and non-clastic rocks, there might be a possibility or an increased porosity. This increase in porosity will be a result of dissolution or mineralogical changes occurring in the original composition of the sediments.^76^

Spatial and vertical variations of the carbonate reservoir quality in the Balkassar oil field reveal the effects of compaction on porosity and pressure. The integrated approach of seismic and well data is substantial in defining the reservoir properties.^64^ The results of both the data sets indicate tight formation conditions in the Sakesar Limestone with the total/average porosity values ranging between 2 and 4% (Figure 5). This suggests that the overburden pressure increased, resulting in porosity reduction and fluid expulsion, as the deposition continues.^54^ If the sedimentation rate is slow, rocks are compacted in the normal compaction trend, with maintaining the equilibrium between the increasing overburden and fluid expulsion.^53^

Sakesar is a proven reservoir in the Potwar Basin,^44^ but the formation is mostly water-saturated and has poor reservoir capability in terms of fluid accommodation. Porosity is one of the most important factors that determine whether or not a petroleum accumulation may be economically viable.^1^ The effective porosity also indicates that the formation is highly compacted having values averaging at about 1–2% in most of its thickness. Only a 10 m bottommost part of the formation at the depth of around 2590 m showed an appreciable amount of porosity values ranging between 7 and 8%. This lower part of the formation shows a deviation from the normal compaction trend where porosity might have been retained due to nonexpulsion of the fluid.^26^

After evaluating the porosities, PP is predicted with the help of the sonic log-based Eaton’s method,^52,77^ which indicates mostly underpressure conditions in the Sakesar Limestone. A few peaks representing overpressure zones are marked at the shallower depths, whereas the major overpressured zone is identified at the base of the formation (Figure 6). This indicates that most of the fluid present in the rock had enough time to escape out of it, to maintain equilibrium with overburden pressure. A slow and steady depositional environment supported the pore fluids to squeeze out during the deposition.^7^

Changes in the porosity and pressure values with respect to time and burial depths have been evaluated using the burial history plot. As depth is not the only factor controlling the rock porosity, vertical effective stress in response of overburden pressure also contributes to it.^14^ The burial history plot of the Sakesar Limestone shows two episodes of subsidence during its depositional history. Both events are marked by the Indian and Eurasian post-collisional depositional changes during the Miocene times. Uplifting of the Indian plate was followed by the high sedimentation influx of the molasse sediment, which resulted in an increase of lithostatic pressure.^53^ A high subsidence rate in response of the increased sediment influx could possibly cause the overpressuring in the Eocene carbonates. Variations of the sediment influx during the Miocene times also affected the reservoir quality of Eocene carbonates. This resulted in escalation of the porosity reduction rate and increase in pore pressure values at the base of the formation (Figure 7a,b).

As the well-based porosities indicate one-dimensional vertical porosity variations, seismic porosities are also generated to evaluate the vertical and lateral variations throughout the study area. Integrating geology, petrophysical, and geophysical data through seismic inversion has improved the reservoir assessment.^67^ Seismic porosities are estimated with the help of the PNN technique on the whole seismic cube.^78,79^ Results indicate that the formation bears extremely low porosities throughout the study area (Figures 13 and 14). These low porosity values justify the high porosity reduction rates determined through the geohistory analysis. The northern part of the Balkassar oil field, around the Balkassar 01A well, indicates extremely tight conditions, having less than 2% porosity; however, at the Balkassar OXY 01, values are as high as 4%.

## 6 Conclusions

Compaction in younger Tertiary basins, dominated by variable deposition rates, plays a critical role in the development of carbonate reservoir properties. Multiple episodes of rapid deposition affect the mechanical compaction of the Sakesar Limestone of the Eocene age. The compaction trend analysis revealed that these massive carbonate beds mostly followed a normal compaction trend at shallow depths under mechanical compaction. The prevailing underpressure conditions depict that the rock is dewatered freely with progressive burial depth apart from a 10 m interval at the base of the formation. Vertical and lateral porosity variations computed with the help of log and seismic porosities represent tight formation conditions with average values ranging between 2 and 4%. These massive limestone units had enough time for the pore fluid to squeeze out in response to the increasing overburden pressure, which resulted in degradation of its reservoir quality.

## Notes

M.H.was employed by the company LMK Resources, Islamabad, 44000, Pakistan. The remaining authors declare that the research was conducted in the absence of any commercial or financial relationships that could be construed as a potential conflict of interest.

## References

[ref1] Worden R.; et al. Petroleum reservoir quality prediction: overview and contrasting approaches from sandstone and carbonate communities. Geol. Soc. Spec. Publ. 2018, 435, 1–31. 10.1144/sp435.21.

[ref2] Lai J.; Liu S.; Xin Y.; et al. Geological-petrophysical insights in the deep Cambrian dolostone reservoirs in Tarim Basin, China. AAPG Bull. 2021, 105, 2263–2296. 10.1306/03122119135.

[ref3] Yousef I.; Morozov V.; Kolchugin A.; et al. Impact of microfacies and diagenesis on the reservoir quality of Upper Devonian carbonates in Southeast Tatarstan, Volga-Ural Basin, Russia. Pet. Res. 2022, 10.1016/j.ptlrs.2022.10.006.

[ref4] Wahid A.; et al. Impact of complex tectonics on the development of geo-pressured zones: A case study from petroliferous Sub-Himalayan Basin, Pakistan. Geopersia 2022, 12, 89–106. 10.22059/geope.2021.324799.648618.

[ref5] Bredesen K.; Avseth P.; Johansen T. A.; et al. Rock physics modelling based on depositional and burial history of Barents Sea sandstones. Geophysical Prospecting 2019, 67, 825–842. 10.1111/1365-2478.12683.

[ref6] Dasgupta T.; Mukherjee S. Sediment Compaction and Applications in Petroleum Geoscience; Springer, 2020.

[ref7] Abbey C. P.; Osita M. C.; Sunday O. A.; et al. Disequilibrium Compaction, Fluid expansion and unloading effects: Analysis from well log and its pore pressure implication in Jay Field, Niger Delta. Iraqi J. Sci. 2020, 389–400. 10.24996/ijs.2020.61.2.17.

[ref8] Mujtaba M. Source Rock Distribution and Evaluation in the Middle Indus Basin Pakistan; Hydrocarbon Development Institute of Pakistan, R & D project report, 1999; p 161.

[ref9] Croizé D.; Renard F.; Gratier J.-P. Compaction and porosity reduction in carbonates: A review of observations, theory, and experiments. Adv. Geophys. 2013, 54, 181–238.

[ref10] Coogan A.; Manus R. Chapter 3 Compaction and Diagenesis of Carbonate Sands. In Developments in Sedimentology; Elsevier, 1975; pp 79–166.

[ref11] Bjørlykke K. Modelling of Sediment Compaction During Burial in Sedimentary Basins, in Elsevier Geo-Engineering Book Series; Elsevier, 2004; pp 699–708.

[ref12] Ricken W. The carbonate compaction law: a new tool. Sedimentology 1987, 34, 571–584. 10.1111/j.1365-3091.1987.tb00787.x.

[ref13] Yasin Q.; Sohail G. M.; Khalid P.; et al. Application of machine learning tool to predict the porosity of clastic depositional system, Indus Basin, Pakistan. J. Pet. Sci. Eng. 2021, 197, 107975 10.1016/j.petrol.2020.107975.

[ref14] Zhang J. Effective stress, porosity, velocity and abnormal pore pressure prediction accounting for compaction disequilibrium and unloading. Mar. Pet. Geol. 2013, 45, 2–11. 10.1016/j.marpetgeo.2013.04.007.

[ref15] Bjorlykke K. Sediment compaction and rock properties. In AAPG iNternational Conference and Exhibition; Citeseer, 2008.

[ref16] Chen J.; Kuang X.; Zheng C. An empirical porosity–depth model for Earth’s crust. Hydrogeol. J. 2020, 28, 2331–2339. 10.1007/s10040-020-02214-x.

[ref17] Sajed O. K. M.; Glover P. W. Dolomitisation, cementation and reservoir quality in three Jurassic and Cretaceous carbonate reservoirs in north-western Iraq. Mar. Pet. Geol. 2020, 115, 104256 10.1016/j.marpetgeo.2020.104256.

[ref18] Haghighat A.; et al. Depositional and diagenetic impact on reservoir quality of the Asmari carbonate reservoir, Naft-Sefid Oilfield, SW Iran. Geopersia 2021, 11, 219–243. 10.22059/GEOPE.2020.309894.648576.

[ref19] Neugebauer J. Some aspects of cementation in chalk. Pelagic Sediments: on Land and Under the Sea 1974, 1, 149–176.

[ref20] Schmoker J. W.; Halley R. B. Carbonate porosity versus depth: a predictable relation for south Florida. AAPG Bull. 1982, 66, 2561–2570. 10.1306/03B5AC73-16D1-11D7-8645000102C1865D.

[ref21] Boutaleb K.; Baouche R.; Sadaoui M.; et al. Sedimentological, petrophysical, and geochemical controls on deep marine unconventional tight limestone and dolostone reservoir: Insights from the Cenomanian/Turonian oceanic anoxic event 2 organic-rich sediments, Southeast Constantine Basin, Algeria. Sediment. Geol. 2022, 429, 106072 10.1016/j.sedgeo.2021.106072.

[ref22] Scholle P. A. Chalk diagenesis and its relation to petroleum exploration: oil from chalks, a modern miracle?. AAPG Bull. 1977, 61, 982–1009.

[ref23] Ehrenberg S. N.; Nadeau P. Sandstone vs. carbonate petroleum reservoirs: A global perspective on porosity-depth and porosity-permeability relationships. AAPG Bull. 2005, 89, 435–445. 10.1306/11230404071.

[ref24] Bjorlykke K. Petroleum Geoscience: from Sedimentary Environments to Rock Physics; Springer Science & Business Media, 2010.

[ref25] Bowers G. L. Pore pressure estimation from velocity data: Accounting for overpressure mechanisms besides undercompaction. SPE Drill. Completion 1995, 10, 89–95. 10.2118/27488-PA.

[ref26] Udo K.; Akpan M.; Agbasi O. Estimation of Overpressures in Onshore Niger Delta Using Wire-line Data. Int. J. Sci. Res. 2015, 4, 2780–2784.

[ref27] Law B. E.; Shah S. H. A.; Malik M. A. Memoir 70, Chapter 14: Abnormally High Formation Pressures, Potwar Plateau, Pakistan,1998.

[ref28] Hart B.; Flemings P.; Deshpande A. Porosity and pressure: Role of compaction disequilibrium in the development of geopressures in a Gulf Coast Pleistocene basin. Geology 1995, 23, 45–48. 10.1130/0091-7613(1995)023<0045:PAPROC>2.3.CO;2.

[ref29] Warwick P. D. Overview of the geography, geology and structure of the Potwar regional framework assessment project study area, north-ern Pakistan Regional Studies of the Potwar Plateau Area, Northern Pakistan. US Geol. Surv. Bull; Warwick P. D., Wardlaw B. R.; U.S. Agency for International Development, 2007, 2078.

[ref30] Aamir M.; Siddiqui M. M. Interpretation and visualization of thrust sheets in a triangle zone in eastern Potwar, Pakistan. The Leading Edge 2006, 25, 24–37. 10.1190/1.2164749.

[ref31] Amjad M. R.; et al. Petrophysical and Geochemical Analysis of Chichali Formation for the Source Rock Evaluation: A Case Study of Chanda-01 Well, Upper Indus Basin, Pakistan. Int. J. Econ. Environ. Geol. 2019, 32–39.

[ref32] Khan M.; et al. Geology of petroleum in Kohat-Potwar depression, Pakistan. AAPG Bull. 1986, 70, 396–414.

[ref33] Ashraf U.; et al. Analysis of Balkassar area using velocity modeling and interpolation to affirm seismic interpretation Upper Indus Basin. Geosciences 2016, 6, 78–91.

[ref34] Baker D. M.; et al. Development of the Himalayan frontal thrust zone: Salt Range, Pakistan. Geology 1988, 16, 3–7. 10.1130/0091-7613(1988)016<0003:DOTHFT>2.3.CO;2.

[ref35] Jaumé S. C.; Lillie R. J. Mechanics of the Salt Range-Potwar Plateau, Pakistan: A fold-and-thrust belt underlain by evaporites. Tectonics 1988, 7, 57–71. 10.1029/TC007i001p00057.

[ref36] Pennock E. S.; et al. Structural interpretation of seismic reflection data from eastern Salt Range and Potwar Plateau, Pakistan. AAPG Bull. 1989, 73, 841–857.

[ref37] Raza H. A.; et al. Petroleum zones of Pakistan. Pak. J. Hydrocarbon Res. 1989, 1, 1–20.

[ref38] Gee E. R.; Gee D. Overview of the geology and structure of the Salt Range, with observations on related areas of northern Pakistan. Geol. Soc. Am. Spec. Pap. 1989, 232, 95–112.

[ref39] Shakir U.; et al. Structural Delineation and Hydrocarbon Potential Evaluation of Lockhart Limestone in Basal Area, Upper Indus Basin, Pakistan. Nucleus 2019, 56, 55–62.

[ref40] Iqbal S.; Akhter G.; Bibi S. Structural model of the Balkassar area, Potwar Plateau, Pakistan. International Journal of Earth Sciences 2015, 104, 2253–2272. 10.1007/s00531-015-1180-4.

[ref41] Stoneley R. Evolution of the Continental Margins Bounding a Former Southern Tethys, in the Geology of Continental Margins; Springer, 1974; pp 889–903.

[ref42] Lillie R. J. Structural development within the Himalayan foreland fold-and-thrust belt of Pakistan. Sedimentary Basins and Basin-Forming Mechanisms—Memoir 12; CSPG Special Publications, 1987; pp 379–392.

[ref43] Awais M.; Hanif M.; Jan I. U.; et al. Eocene carbonate microfacies distribution of the Chorgali Formation, Gali Jagir, Khair-e-Murat Range, Potwar Plateau, Pakistan: approach of reservoir potential using outcrop analogue. Arabian J. Geosci. 2020, 13, 1–18. 10.1007/s12517-020-05377-9.

[ref44] Ishaq M.; Jan I. U.; Hanif M.; et al. Microfacies and diagenetic studies of the early Eocene Sakesar Limestone, Potwar Plateau, Pakistan: approach of reservoir evaluation using outcrop analogue. Carbonates Evaporites 2019, 34, 623–656. 10.1007/s13146-018-0430-5.

[ref45] Wrobel-Daveau J.-C. Insights on Fractured Domains in Reservoirs Resulting from Modeling Complex Geology/Structures-Case Study of the Ratana Field in the Potwar Basin, Pakistan. In SPE Middle East Oil & Gas Show and Conference; OnePetro, 2021.

[ref46] Fahad M.; Khan M. A.; Hussain J.; et al. Microfacies analysis, depositional settings and reservoir investigation of Early Eocene Chorgali Formation exposed at Eastern Salt Range, Upper Indus Basin, Pakistan. Carbonates Evaporites 2021, 36, 1–18. 10.1007/s13146-021-00708-7.

[ref47] Shah S. Stratigraphy of Pakistan: GSP memoir 22; Geological Survey of Pakistan: Quetta, 2009.

[ref48] Moghal M. A.; et al. Subsurface geometry of Potwar sub-basin in relation to structuration and entrapment. Pak. J. Hydrocarbon Res. 2007, 17, 61–72.

[ref49] Jadoon I. A.; Hinderer M.; Wazir B.; et al. Structural styles, hydrocarbon prospects, and potential in the Salt Range and Potwar Plateau, north Pakistan. Arabian J. Geosci. 2015, 8, 5111–5125. 10.1007/s12517-014-1566-9.

[ref50] Rider M. The Geological Interpretation of Well Logs, 2nd ed.; Rider-French Consulting Ltd.: Scotland, 1996.

[ref51] Radwan A. E.; Sen S. Characterization of in-situ stresses and its implications for production and reservoir stability in the depleted El Morgan hydrocarbon field, Gulf of Suez rift basin, Egypt. J. Struct. Geol. 2021, 148, 104355 10.1016/j.jsg.2021.104355.

[ref52] Eaton B. A. The equation for geopressure prediction from well logs. In Fall Meeting of the Society of Petroleum Engineers of AIME; OnePetro, 1975.

[ref53] Amjad M. R.; Zafar M.; Ahmad T.; et al. Overpressures Induced by Compaction Disequilibrium Within Structural Compartments of Murree Formation, Eastern Potwar, Pakistan. Front. Earth Sci. 2022, 10, 903405 10.3389/feart.2022.903405.

[ref54] Yassir N.; et al. Relationships between pore pressure and stress in different tectonic settings. Mem-Am- Assoc. Pet. Geol. 2002, 79–88.

[ref55] Wang Z.; Wang R. Pore pressure prediction using geophysical methods in carbonate reservoirs: Current status, challenges and way ahead. J. Nat. Gas Sci. Eng. 2015, 27, 986–993. 10.1016/j.jngse.2015.09.032.

[ref56] Nwankwo C. N.; Kalu S. O. Integrated approach to pore pressure and fracture pressure prediction using well logs: case study of onshore Niger-Delta sedimentary Basin. Open J. Geol. 2016, 06, 1279–1295. 10.4236/ojg.2016.610094.

[ref57] Batzle M.; Wang Z. Seismic properties of pore fluids. Geophysics 1992, 57, 1396–1408. 10.1190/1.1443207.

[ref58] Biot M. A. General theory of three-dimensional consolidation. J. Appl. Phys. 1941, 12, 155–164. 10.1063/1.1712886.

[ref59] Terzaghi K.; Peck R. B.; Mesri G. Soil Mechanics in Engineering Practice; John Wiley & Sons: 1996.

[ref60] Zhang J. J. Applied Petroleum Geomechanics; Gulf Professional Publishing, 2019.

[ref61] Haq B. U.; Hardenbol J.; Vail P. R. Chronology of fluctuating sea levels since the Triassic. Science 1987, 235, 1156–1167. 10.1126/science.235.4793.1156. 17818978

[ref62] Wangen M.; Antonsen B.; Fossum B.; et al. A model for compaction of sedimentary basins. Appl. Math. Modell. 1990, 14, 506–517. 10.1016/0307-904X(90)90183-6.

[ref63] Hampson D. P.; Russell B. H.; Bankhead B. Simultaneous Inversion of Pre-stack Seismic Data, SEG Technical Program Expanded Abstracts 2005, Society of Exploration Geophysicists, 2005; pp 1633–1637.

[ref64] Soleimani F.; Hosseini E.; Hajivand F. Estimation of reservoir porosity using analysis of seismic attributes in an Iranian oil field. J. Pet. Explor. Prod. Technol. 2020, 10, 1289–1316. 10.1007/s13202-020-00833-4.

[ref65] Barclay F.; et al. Seismic inversion: Reading between the lines. Oilfield Rev. 2008, 20, 42–63.

[ref66] Ashraf U.; Zhang H.; Anees A.; et al. A core logging, machine learning and geostatistical modeling interactive approach for subsurface imaging of lenticular geobodies in a clastic depositional system, SE Pakistan. Nat. Resour. Res. 2021, 30, 2807–2830. 10.1007/s11053-021-09849-x.

[ref67] Bashir Y.; Faisal M. A.; Biswas A.; et al. Seismic expression of miocene carbonate platform and reservoir characterization through geophysical approach: application in central Luconia, offshore Malaysia. J. Pet. Explor. Prod. 2021, 11, 1533–1544. 10.1007/s13202-021-01132-2.

[ref68] Ashraf U.; Zhang H.; Anees A.; et al. Controls on reservoir heterogeneity of a shallow-marine reservoir in Sawan Gas Field, SE Pakistan: Implications for reservoir quality prediction using acoustic impedance inversion. Water 2020, 12, 2972 10.3390/w12112972.

[ref69] Adekanle A.; Enikanselu P. Porosity prediction from seismic inversion properties over ‘XLD’Field, Niger Delta. Am. J. Sci. Ind. Res. 2013, 4, 31–35. 10.5251/ajsir.2013.4.1.31.35.

[ref70] Ehsan M.; Gu H. An integrated approach for the identification of lithofacies and clay mineralogy through Neuro-Fuzzy, cross plot, and statistical analyses, from well log data. J. Earth Syst. Sci. 2020, 129, 101 10.1007/s12040-020-1365-5.

[ref71] Qiang Z.; Yasin Q.; Golsanami N.; et al. Prediction of Reservoir Quality from Log-Core and Seismic Inversion Analysis with an Artificial Neural Network: A Case Study from the Sawan Gas Field, Pakistan. Energies 2020, 13, 486 10.3390/en13020486.

[ref72] Bjørlykke K. Principal aspects of compaction and fluid flow in mudstones. Geol. Soc. Spec. Publ. 1999, 158, 73–78. 10.1144/gsl.sp.1999.158.01.06.

[ref73] Puttiwongrak A.; Giao P. H.; Vann S. An easily used mathematical model of porosity change with depth and geologic time in deep shale compaction. GEOMATE J. 2020, 19, 108–115. 10.21660/2020.73.39179.

[ref74] Schneider F.; Potdevin J.; Wolf S.; et al. Mechanical and chemical compaction model for sedimentary basin simulators. Tectonophysics 1996, 263, 307–317. 10.1016/S0040-1951(96)00027-3.

[ref75] Bjørlykke K.; Ramm M.; Saigal G. C. Sandstone diagenesis and porosity modification during basin evolution. Geologische Rundschau 1989, 78, 243–268. 10.1007/BF01988363.

[ref76] Mazzullo S.; Harris P. An Overview of Dissolution Porosity Development in the Deep-burial Environment, with Examples from Carbonate Reservoirs in the Permian Basin; West Texas Geological Society: Midland, TX, 1991; pp 125–138.

[ref77] Zhang J. Pore pressure prediction from well logs: Methods, modifications, and new approaches. Earth-Science Reviews 2011, 108, 50–63. 10.1016/j.earscirev.2011.06.001.

[ref78] Din N. U.; Hongbing Z. Porosity prediction from model-based seismic inversion by using probabilistic neural network (PNN) in Mehar Block, Pakistan. Episodes J. Int. Geosci. 2020, 43, 935–946. 10.18814/epiiugs/2020/020055.

[ref79] Ashraf U.; et al. Estimation of porosity and facies distribution through seismic inversion in an unconventional tight sandstone reservoir of Hangjinqi area, Ordos basin. Front. Earth Sci. 2022, 10, 1014052 10.3389/feart.2022.1014052.

